# Yki/YAP, Sd/TEAD and Hth/MEIS Control Tissue Specification in the *Drosophila* Eye Disc Epithelium

**DOI:** 10.1371/journal.pone.0022278

**Published:** 2011-07-19

**Authors:** Tianyi Zhang, Qingxiang Zhou, Francesca Pignoni

**Affiliations:** 1 Department of Ophthalmology, Center for Vision Research, and SUNY Eye Institute, SUNY Upstate Medical University, Syracuse, New York, United States of America; 2 Departments of Biochemistry and Molecular Biology, and Neuroscience and Physiology, SUNY Upstate Medical University, Syracuse, New York, United States of America; University of Texas MD Anderson Cancer Center, United States of America

## Abstract

During animal development, accurate control of tissue specification and growth are critical to generate organisms of reproducible shape and size. The eye-antennal disc epithelium of *Drosophila* is a powerful model system to identify the signaling pathway and transcription factors that mediate and coordinate these processes. We show here that the Yorkie (Yki) pathway plays a major role in tissue specification within the developing fly eye disc epithelium at a time when organ primordia and regional identity domains are specified. RNAi-mediated inactivation of Yki, or its partner Scalloped (Sd), or increased activity of the upstream negative regulators of Yki cause a dramatic reorganization of the eye disc fate map leading to specification of the entire disc epithelium into retina. On the contrary, constitutive expression of Yki suppresses eye formation in a Sd-dependent fashion. We also show that knockdown of the transcription factor Homothorax (Hth), known to partner Yki in some developmental contexts, also induces an ectopic retina domain, that Yki and Scalloped regulate Hth expression, and that the gain-of-function activity of Yki is partially dependent on Hth. Our results support a critical role for Yki- and its partners Sd and Hth - in shaping the fate map of the eye epithelium independently of its universal role as a regulator of proliferation and survival.

## Introduction

Animal body size and shape is genetically determined by evolutionarily conserved signaling pathways that control patterning and growth. The activity of these pathways is integrated to produce organisms of similar dimensions and body pattern for a given biological species. How different signaling pathways contribute to these processes is intensely studied in the imaginal discs of *Drosophila*, including the eye-antennal disc epithelium. Although there is significant overlap in the control of tissue specification and growth within the larval eye epithelium, a primary role can be ascribed to most signaling pathways such that they contribute in a predominant fashion to either patterning or proliferation.

For instance, changes in the level or distribution of the secreted morphogen Decapentaplegic (Dpp), a BMP 2/4 type factor, affects proliferation within the disc. Indeed, Dpp acts as either an inducer or a suppressor depending on timing, spatial distribution and likely concentration (reviewed [1]). However, the most striking effect of changes in Dpp expression is on the early fate map with dramatic changes in the location, size, and even ectopic gain of organ primordial [2]–[5].

Conversely, the Hippo -Yorkie (Hpo-Yki) signaling pathway is known as a virtually universal regulator of proliferation and survival in both vertebrates and invertebrates (reviewed in [6]–[10]). In *Drosophila*, it is thought to function in essentially all tissues as a major regulator of organ and body size. In agreement with this view, it has been shown to regulate cell proliferation and survival in the larval eye disc as well [11]–[14], but has not been implicated in the early patterning of this tissue. The core transduction elements of the pathway include several kinases that control the cytoplasmic versus nuclear localization of the non-DNA-binding transcriptional co-activator protein called Yki in flies or YES-Associated-Protein (YAP) in vertebrates. These include the kinase Hpo which phosphorylates and activates the kinase Warts (Wts). Phosphorylated Wts, then, associates with the protein Mob-as-tumor-suppressor (Mats) to, in turn, phosphorylate Yki. Phosphorylated Yki is sequestered in the cytoplasm, effectively preventing it from controlling gene expression. Thus, in the presence of Hpo signaling, Yki is inactive. Conversely, in the absence of Hpo signaling, Yki is active, nuclear, and associates with DNA-binding partners to form complexes that regulate gene expression.

One direct partner is the TEAD/TEF-type DNA-binding protein Scalloped (Sd). Sd and Yki contribute to the up-regulation of the genes *Diap1, Bantam* and *CyclinE*, thereby promoting cell survival and proliferation in the developing wing epithelium [12]–[13], [15]. In the developing eye, however, though Yki is clearly critical to proliferation, Sd appears to make a minor contribution [14]. Recently, the transcription factors Homothorax (Hth), a TALE-type homeobox protein, and Teashirt, a zinc finger protein, have been shown to associate with Yki and contribute to the up-regulation of the pro-proliferation microRNA *Bantam* in eye progenitor cells [14].

**Figure 1:**
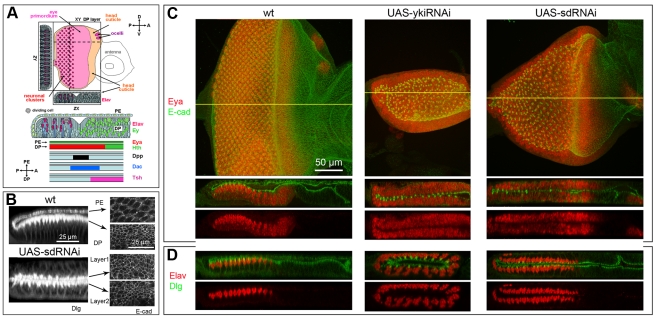
Loss of Yki or Sd changes the fate map of the eye disc. In all figures: posterior is on left; Gal4 driver is Act>IC>Gal4 unless otherwise stated; UAS transgenes used are as marked in panels; magnification shown in first panel applies to all panels in figure unless otherwise marked; XY images show projections of all Z optical planes unless otherwise stated; ZX and ZY images of disc scans were edited to display the selected regions of the discs marked by yellow lines in the XY view; colors have been chose to optimize image quality and do not always reflect the corresponding detection channel; for precise genotypes and temperature of crosses see Table S1. A) Schematic diagram of developing eye disc at the L3 stage with expression pattern of molecular markers used in this work. Larger image is provided in Fig. S1A. B) In the wt (top), PE cells are squamous and DP cells are columnar. In sd-RNAi-expressing discs (bottom), both cell layers display the columnar appearance of the pseudostratified DP monolayer. As shown by the uneven staining of the apical versus basal portions of the cell membranes and the position of the neuronal nuclei shown in panel D, the polarity of the cells is not disrupted; the apical side faces the disc lumen in all cases. C, D) wt (left), yki-RNAi (middle) and sd-RNAi (right) discs; C) L3 discs stained for the RDF Eya (red) to show retinal cells and the apical membrane marker E-cadherin (green) to highlight cell morphology. Mutant discs display two DP-like retina forming cell layers. D) L3 discs stained for the neuronal marker Elav (red) and the membrane marker Dlg (green). Mutant discs contain developing mirror-image, neuronal arrays; in both cell layers, neurons begin to form at the posterior and rows are added progressively expanding the ommatidial field towards the anterior.

The eye-antennal disc is a powerful model system for studying the genetic control of both proliferation and tissue specification. It gives rise to both neural (including several sensory organs) and non-neural fly head structures. The ‘eye’ portion of the epithelium (called eye disc) consists of a sheet of cells that gives rise to eye, ocelli (additional light-sensory organs) and cuticle of the fly head. The developing epithelium is folded into a flattened sac with two opposing cell layers separated by a lumen but continuous along much of the disc margin (Figs. 1A, S1A). During the L1 and L2 larval stages, the eye disc grows through proliferation and acquires regional identity. By the last larval stage (L3), the two layers can be readily distinguished by morphology and, within them, groups of cells are already fated to give rise to defined regions of the adult fly head (Figs. 1A, S1A). The disc proper (DP) cell layer has columnar, pseudostratified morphology, and is known to give rise to the adult eye and surrounding cuticle. The squamous peripodial portion of the epithelium (PE) is much less well understood and contributes to cuticle of the ventral and posterior regions of the fly head. Differences in morphology between the two cell layers can first be detected early in L2 (reviewed in [16]). During the L2 stage, the transcription factors Eyeless (Ey), Teashirt (Ths), Eyes absent (Eya), Sine oculis (So), and Dachshund (Dac), collectively called Retina Determination Factors (RDFs), come to be co-expressed (Ey, Tsh –>Eya –>So –>Dac) within a portion of the DP and hence define the eye or retina organ primordium. At the L3 stage, a wave of secreted factors sweeps across the DP cell layer from posterior to anterior and induces eye progenitors to stop dividing, acquire a specific cell fate and begin to differentiate into the photoreceptor neurons and accessory cells that form each single eye or ommatidium (Figs. 1A, S1A) (reviewed in [17]–[18]). Recent studies have shown that the PE contributes to the development of the DP cell layer, to the formation of several adult head structures and to the reorganization of the discs during metamorphosis (reviewed in [16]). Despite this, our understanding of the genetic control of specification, cell morphology and differentiation in the PE is still in its infancy.

In this paper we identify three major players (Yki, Sd and Hth) in the development of the PE. We show that these transcriptional regulators control regional specification of the PE by blocking formation of an ectopic retina organ primordium within this cell layer and thus promoting PE/cuticle formation. In addition, we implicate upstream regulators of Yki within the Hippo signaling pathway in the proper patterning of the eye disc. We also show that gene function is required in the PE and at the time of regional specification (L2 stage) to promote PE identity. We propose that these activities reflect a function of the Hpo/Yki pathway in tissue specification that is distinct from its activity in regulating tissue growth and survival, and that partner-specificity, in addition to Yki activity levels, contributes in a significant way to the differential activity of Yki in specification versus proliferation in different regions of the eye disc.

## Results

### Loss of Yki or Sd function causes the transformation of the PE into DP/retina

In the course of an RNAi-based genetic screen [Zhang & Pignoni, unpublished] to identify early players in the patterning and development of the fly eye (*ey-Flip; Act>IC>Gal4 UAS-GFP; UAS-Dicer2/UAS-RNAi*; see Fig. S1B and legend for driver expression pattern), we discovered that the disc-wide knock-down of Yki or its partner Sd induced a dramatic reorganization of the eye-disc fate map (here and elsewhere refer to Table S1 for exact genotypes). Instead of one squamous (PE) and one columnar (DP) cell layer, all sd-RNAi- or yki-RNAi-expressing L3 discs displayed 2 columnar DP-like layers (Fig. 1B, not shown). The yki-RNAi and sd-RNAi discs were considerably smaller than similar staged wild type (wt) discs and both cell layers strongly expressed the RD factor Eya, a marker for retinal progenitors as well as developing retinal cells (Fig. 1C). In addition, cells expressing the pan-neural marker Elav were seen not only in the DP layer (as in the wt) but also in the transformed PE, indicating that neurogenesis and ommatidia assembly had occurred in both cell layers (Fig. 1D). We confirmed that Yki protein expression was, in fact, down-regulated by this yki-RNAi line in clones (Fig. S2A) and that similar phenotypes were induced by other, previously published, RNAi-transgenes for *yki* and *sd* (Table S2, Fig. S3A, B) [13]. Thus, we believe that the changes we detected in disc morphology and fate are indeed the product of down-regulation of Yki and Sd expression.

Surprisingly, previous studies of Yki and Sd in the eye disc have not uncovered a function in cell fate [11]–[14]. Loss-of-function of *yki*, in particular, has been reported to affect cell proliferation and survival profoundly but not cell fate even when one of the same markers we used, the neuronal marker Elav, was used to assess development of mutant clones [14]. In three of these cases traditional loss-of-function mutant clones, with and without Minute, were generated [11]–[12], [14]. In one case, a similar approach to ours was utilized, *sd* or *yki* were silenced through RNAi [13]. To investigate the source of this discrepancy, we also generated sd-RNAi- or yki-RNAi-expressing clones by the MARCM method. As expected, RNAi-expressing clones were reduced in size as compared to GFP-only control clones in both cases, though much more severely in the case of yki-RNAi (Fig. S4). In neither case, did we detect any change in fate; regardless of whether the clones laid within the DP or PE cell layer (Fig. S4). These results are consistent with the previously reported clonal phenotypes and show that differences in the gene knock-out approaches (rather than the use of mutant alleles as opposed to RNAi transgenes) are responsible for the discrepancy in mutant phenotypes. As explained in the discussion, these techniques differ in two ways: the extent of mutant tissue generated and the timing of gene function inactivation, pointing to either non-cell-autonomy or a early (L1/L2) gene function as explanations for these observations.

In conclusion, loss of Yki or Sd function results in a striking transformation of the PE cell layer into a second DP with a developing retina (PE-to-PD/retina transformation), indicating that the activity of these proteins is required for the establishment of PE identity.

### Yki function is required in the PE to establish PE-fate

**Figure 2:**
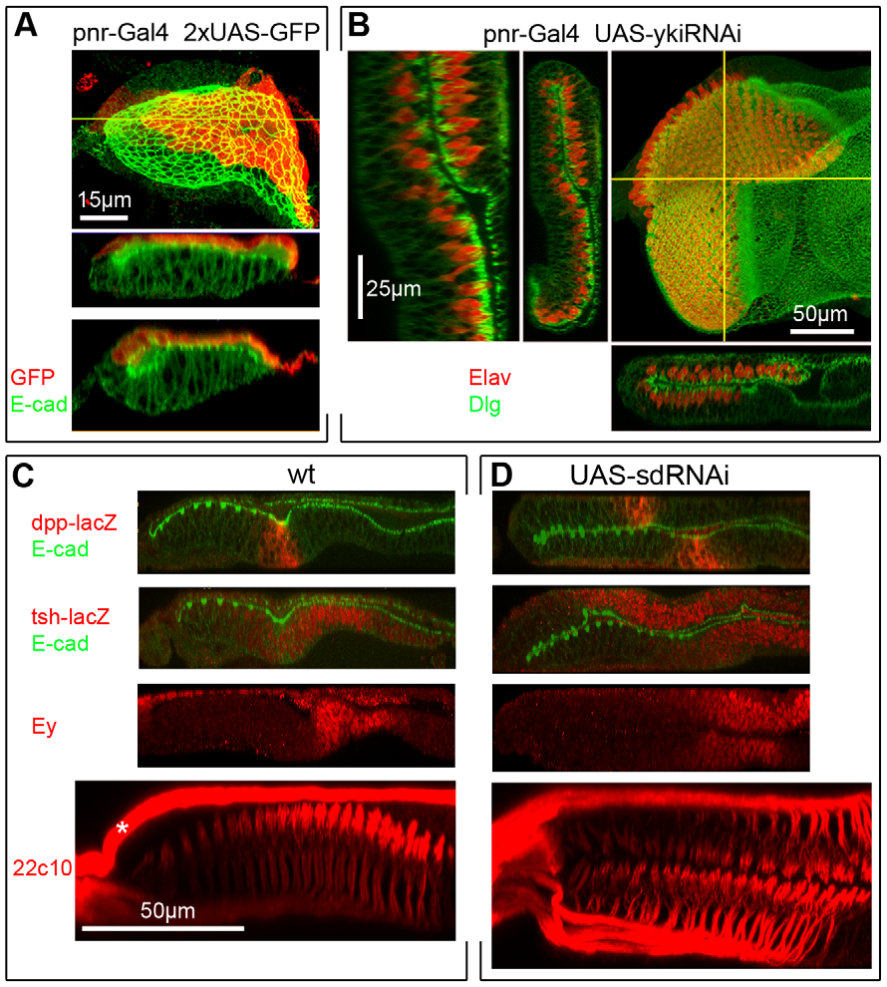
Gene silencing in the PE is sufficient to induce the PE–to-DP transformation. A) *pnr-Gal4* drives expression in ∼early-mid L2 discs. Top: XY and ZX views of one disc; bottom: ZX of another. Gal4 is expressed in a broad domain within the dorsal PE. B) *pnr-Gal4 UAS-ykiRNAi* disc stained for Dlg (for cell shape) and Elav (for neurons). Right: confocal XY image of L3 disc with ZX and ZY projections; left: high magnification ZY of the same disc. PE-restricted expression of yki-RNAi transforms the dorsal region of the PE into DP/retina. High magnification panel shows that the transition from transformed to non-transformed region occurs over a few cell diameters. C, D) L3 discs stained in red for (top to bottom) *dpp* (*dpp-lacZ*) which marks the transition zone between eye progenitor cells and developing neurons, the RDFs *tsh* (*tsh-lacZ)* and Ey which mark eye progenitor cells, and the pan-neural membrane marker 22C10 which highlights differentiating neurons and their axons. Where shown, the membrane marker E-cadherin is in green. C) wt L3 discs show normal expression of *dpp-lacZ* in the transition zone where morphogenesis of the ommatidial array begins (visible as a depression in the DP of the wt discs, but not so marked in most transformed discs), *tsh-lacZ* and Ey in eye progenitor cells in the anterior portion of the disc (expression of Ey in the PE layer is also seen), and 22C10 stained neurons projects their axons posteriorly, along the basal side of the DP into the optic stalk and brain (not shown). Asterisk marks an axonal fascicle outside the eye disc (Bolwig's nerve). D) sd-RNAi L3 discs show mirror image duplications of each expression domain in two thicker, DP-like cell layers. The domains of *dpp-lacZ* expression are offset, indicating that, in this disc, neurogenesis in one layer lags behind the other layer. This was not always the case.

Since the *ey-Flip; Act>IC>Gal4* driver drives expression in both PE ad DP cell layers, and *yki* appears to function in a non-cell-autonomous fashion, we sought to investigate whether the transformation phenotype indeed resulted from the loss of Yki function within the PE or involved more complex long range effects. We, therefore, used the PE-specific Gal4 line *pnr-Gal4* [19] to drive either 2 copies of *UAS-GFP* or one copy of a *UAS-yki-RNAi* transgene. In control discs, GFP expression was exclusively restricted to the dorsal side of the PE cell layer throughout the larval stages (Fig. 2A, and not shown). At the L2 stage (when gene function is required, as shown below), GFP was detected in a large dorsal section of the PE cell layer over the entire eye-antennal disc (Fig. 2A). In *pnr-Gal4 yki-RNAi* discs, the dorsal half of the PE epithelium became columnar and displayed a developing ommatidial array by the L3 stage (Fig. 2B). As shown in the magnified panel in Fig. 2B, the transition from the columnar, transformed PE cells to the unchanged squamous PE cells occur sharply within a few cell diameters. Unfortunately, we cannot use this genetic background to explore whether signs of non-cell-autonomy are present because the co-expressed GFP is no longer detected by the stage at which cell fate can be most readily assessed (late L3). This is not unexpected, since the *pnr-Gal4* is a PE-specific driver, hence its expression is down-regulated over time as the transformed PE-cells are locked into a DP fate.

In conclusion, Yki activity is required within the PE cell layer to establish proper tissue identity. For ease of scoring, however, additional experiments were carried out using the *ey-Flip; Act>IC>Gal4* driver that causes transformation of the entire PE.

### Normal development of the ectopic retina in sd-RNAi discs

During normal development, eye neurogenesis initiates at the posterior margin of the L3 disc and expands anteriorly within the DP cell layer, as progressively more anterior rows of eye progenitor cells are induced to begin neuronal differentiation (reviewed in [17], [18]). A stripe of *dpp (dpp-lacz)* expression marks the transition zone wherein groups of progenitor cells commit to a neuronal fate and begin to differentiate (Figs. 1A, 2C). Within the DP, the RDFs Ey and *tsh (tsh-lacZ)* are strongly expressed in progenitor cells ahead of the *dpp* stripe, but absent from the developing ommatidial field posterior to the *dpp*; whereas the neuronal markers Elav and 22C10 mark the differentiating neurons in the posterior region (Figs. 1A, 1D, 2C). The Elav-positive nuclei are located apically within the columnar DP cells, whereas the axons, marked by 22C10, run along the basal side of the disc and extend posteriorly into the optic stalk (Figs. 1C, 2C, S5).

The development of the ectopic retina within the transformed PE follows the same pattern as the normal retina. In sd-RNAi disc, the *dpp*-positive stripe is seen at progressively more anterior positions as the neuronal field expands, thus the stripe sweeps across the disc from posterior to anterior in both cell layers (Fig. 2D). Often but not always, development of the neuronal array in the transformed cell layer lagged behind neurogenesis in the normal DP layer. Ey and *tsh* were strongly expressed in both layers in anterior domains, consistent with expression in retina progenitor cells (Fig. 2D). Whereas expression of the pan-neural markers Elav and 22C10 was observed in the posterior region of each cell layer, as expected for the developing neuronal array. In both layers, Elav positive nuclei were located near the apical (lumenal) surface of the columnar cells, and 22C10 positive axons extended along the basal (external) side towards the posterior margin (Figs. 1D, 2C, 2D, S5).

In summary, the PE-to-DP transformation appears to be complete, at least as it pertains to the retina domain. The anterior-posterior and apical-basal polarity of the transformed tissue is unchanged, and eye morphogenesis begins similarly in both cell layers from the posterior margin of the disc.

### Sd function is required at the L2 stage to suppress retina identity in the PE

The specification of retina primordium within the wt eye disc occurs at the L2 stage, well before eye morphogenesis begins in L3, and at a time when essentially all disc cells are still proliferating. Early in L2, PE and DP cells acquire their distinct morphology [20]. At this time, formation of a retina primordium begins with the induction of Eya expression along the posterior margin of the disc in the DP cell layer [21]. To better understand the process of PE-to-DP/retina transformation, eye discs were analyzed at this earlier developmental stage.

**Figure 3:**
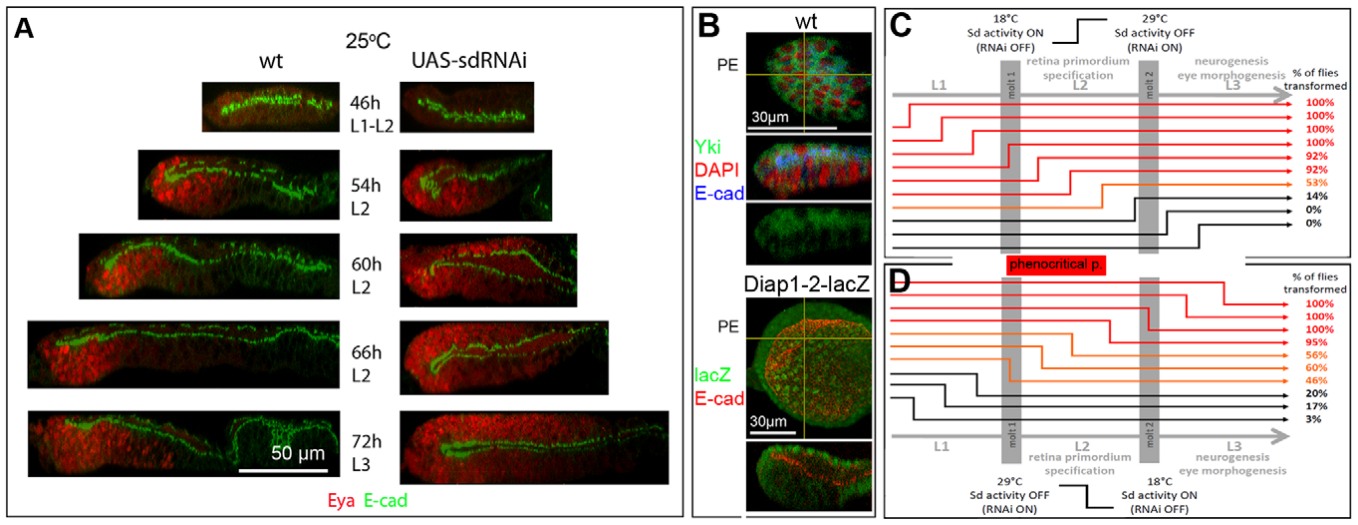
Sd function is required during L2 for PE development. A) ZX view of developing L2 discs stained for Eya (red) and E-cadherin (green). Ectopic Eya expression is clearly seen at 60 hr AEL (∼mid-late L2), but discs are mostly indistinguishable at 54 hr AEL (∼mid L2). B) Top panels: XY and ZX views of wt disc stained for Yki (green), E-cadherin (blue) and DAPI (red). XY shows PE cell layer only. Yki protein is detected throughout the PE and in the apical regions of DP cells. Nuclear Yki is below the detection level of available Ab in the wt eye disc. Bottom panel: XY and ZX views of wt disc stained for ß-galactosidase from the reporter transgene *Diap1-2-lacZ* (green) and E-cadherin (red). XY shows PE cell layer only. Expression of the direct target *Diap1-2-lacZ* can be see in both cell layers and is apparently higher in the PE than the DP during L2, as well as L3 (not shown). C, D) The red bar between graphs marks the phenocritical period in L2. The percentage of flies or pharate adults showing transformation-related phenotypes in each shift experiment is shown to the right. Developmental stage is marked above and below; development proceeds more than twice as fast at 29°C than at 18°C, approximate time of molt between larval stages occurs at different times and is marked by grey columns. Control progeny (maintained at 18°C throughout) develop into normal adults. The lack of a sharp transition is likely due to the indirect method of Sd regulation (Gal80^ts^ = /Gal4 → sd-RNAi = /Sd) which delays changes in Sd protein levels. Time of temperature shift is 12 hr apart in C and 6 hr apart in D. See materials and methods and legend of Fig. S6 for details.

Discs expressing sd-RNAi were indistinguishable from wt until just prior mid-L2 (54 hr AEL 25°C) (Fig. 3A). At this time, the PE cell layer was thinner than the columnar DP in both wt and sd-RNAi discs. At mid-to-late L2 (60 hr AEL 25°C), and more clearly so a few hours later (66 hr AEL 25°C), the PE showed columnar morphology and ectopic Eya expression at the posterior of the disc (Fig. 3A), but the extent of PE-to-DP transformation was somewhat variable from disc to disc. By early L3 (72 h AEL 25°C), all sd-RNAi discs showed strong signs of transformation (Fig. 3A). Thus, the morphological changes and Eya induction associated with PE-to-DP transformation do not become apparent in the L2 stage. This indicates that lack of Sd activity in L2 or earlier results in a failure to maintain PE identity and in a progressive conversion of PE-like tissue into DP. Interestingly, these changes appeared to emanate from the posterior margin of the epithelium (Fig. 3A, S6). This posterior-to-anterior gradient of ectopic eye primordium formation is similar to what is observed during normal development [21]. In wt discs, Eya expression is triggered when cells of the posterior margin begin to secrete Dpp. The similar pattern of primordium development in both cell layers of sd-RNAi discs suggests that Dpp acts as a common inductive signal along the posterior margin.

As mentioned above, developmental analysis of the mutant phenotype indicated that the transformation became apparent in the L2 stage. Yki is, indeed, expressed in L2 eye discs (Fig. 3B) and, although Sd antibodies are not available, expression of a reporter construct under Yki-Sd control (*Diap1*–*2 lacZ;* [12]) is detected in both cell layers at this stage (Fig. 3B). However, RNAi expression under *ey-Flip; Act>IC>Gal4* begins in the embryo, when the eye disc first forms. Hence, the requirement for Yki and Sd in PE formation may reflect protein function at any time prior to the late L2 stage.

To identify the phenocritical period for protein activity, we utilized the Gal4/Gal80^ts^ system [22]. The Gal80^ts^ protein is a temperature sensitive form of the Gal80 negative regulator of Gal4. Thus, at the permissive temperature (18°C), Gal80^ts^ blocks Gal4 activity and expression of the sd-RNAi transgene is prevented (18°C = wt for Sd); whereas at the non-permissive temperature (29°C), Gal80^ts^ is inactive and Gal4 is active, resulting in sd-RNAi transgene expression (29°C = loss-of-function for Sd). Using these reagents, temporal expression of sd-RNAi was controlled by shifting 1 hr embryo collections from 18°C to 29°C, or vice versa, at different time points during larval development. In shift-up (18°C to 29°C) and shift-down (29°C to 18°C) experiments, the extent of PE-to-DP transformation was assessed at the adult stage by scoring eye/head phenotypes that correlated with varying degrees of PE-to-DP transformation in developing discs (see Fig. S6 for detailed approach and data).

Using this approach, we identified the time between the L1/L2 molt and mid-late L2 as the phenocritical period for Sd gene function. As shown in Fig. 3C, the penetrance of transformation-related phenotypes was very high (92% of the flies or more), when *sd* was silenced (shift up experiment) prior to and until mid L2. Gene silencing at mid-late and at the end of L2 resulted in a drastic drop in the number of flies showing signs of transformation, first down to 53% and then to 14%. Later times of *sd* inactivation resulted in no transformation-related phenotypes. Thus, inactivation of endogenous Sd function any time between embryogenesis and about mid-late L2, but not later, compromises tissue identity in the PE indicating that protein activity is required until this stage to lock in the PE fate. Conversely, Fig. 3D shows that restoration of Sd function (shift-down experiment) at or prior to mid-late L1 causes minimal disruption of PE specification (non-transformation phenotypes in ≥80% flies); whereas restoration of Sd function at the L1/L2 molt or thereafter (in L2 and L3) was insufficient to ensure proper PE development (the penetrance of the transformation phenotypes increase from ∼50% of the flies in early L2 to nearly 100% by mid-late L2). Thus, we conclude that Sd function becomes necessary to promote PE identity very early in L2. These results are consistent with the emergence of an Eya-positive ectopic retina domain during the L2 stage as shown in Fig. 3A.

Together, these analyses point to the L2 stage as the time of Sd function in suppressing retina formation and promoting PE identity (Fig. 3C). Noticeably, this is a critical period for eye disc and retina specification within the eye-antennal disc.

### The TALE homeobox factor Hth also contributes to PE development

**Figure 4:**
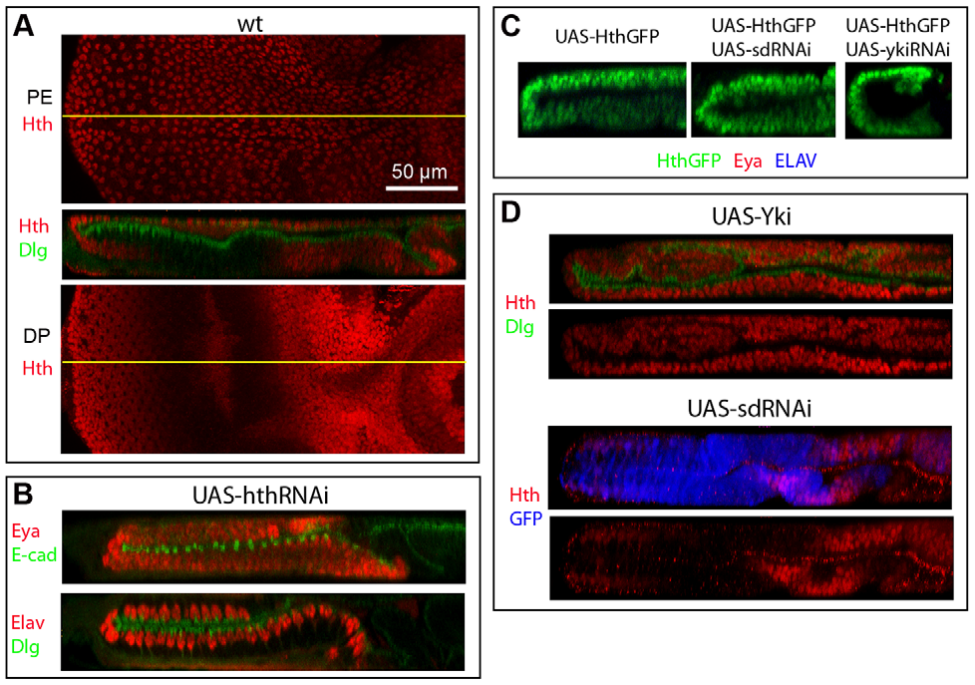
Hth controls PE formation and its expression is regulated by Yki and Sd. A) Wild type L3 disc stained for Hth (red) and Dlg (green); top: XY view of PE layer; middle: XZ view of disc. Bottom: XY view of DP layer. Notice that Hth is broadly expressed in both PE and DP cell layers, but is excluded from part of the eye field. B) ZX views of hth-RNAi expressing discs stained for Eya (red) and E-cadherin (green) (top) or for Elav (red) and Dlg (green) (bottom). Discs show the transformation of PE into a DP with an ectopic retina field (Eya-positive cells) and ectopic neuronal development (Elav-positive clusters). C) ZX views of L3 eye discs expressing an exogenous Hth-GFP fusion protein throughout (left), Hth-GFP plus sd-RNAi (middle), or Hth-GFP plus yki-RNAi (right) stained for anti-GFP (green) to detect Hth-GFP, Eya (red) and Elav (blue). No expression of Eya, or Elav is detected. Co-expression of sd-RNAi or yki-RNAi does not rescue the loss of retina due to Hth mis-expression. D) Top: ZX views of Yki-expressing L3 eye disc stained for Hth (red) and Dlg (green). Over-expression of Yki induces ectopic Hth expression (compare to panel A). Bottom: ZX views of sd-RNAi-expressing L3 eye disc stained for Hth (red) and UAS-GFP (blue). Down-regulation of Sd in cell marked by GFP results in loss of Hth expression. This is consistent with the development of an ectopic retina within the transformed PE as shown in panel B.

Recently, another DNA-binding transcription factor has been implicated in Yki-regulated gene expression. Mann and colleagues have shown that the homeobox protein Homeothorax (Hth) and Yki bind *in vitro* and directly regulate transcription of the gene *Bantam* in eye progenitor cells [14]. Since Hth is also expressed throughout the PE cell layer [23](Fig. 4A) and its mis-expression in the DP can suppress eye morphogenesis (assessed through Eya and Elav expression) [24], [25], we decided to investigate whether Hth plays a role in PE formation.

Knock-down of endogenous Hth was achieved by expressing *UAS-hth-RNAi* transgenes (Table S2, Fig. S2B). Discs were then stained at the L3 stage for retinal and neuronal markers. Interestingly, some hth-RNAi expressing discs displayed two mirror-image DP-like cell layers and no PE, similarly to yki-RNAi expressing discs. Eya and Elav were expressed in both cell layers indicating that two retina primordia had formed and neurogenesis was in progress (Fig. 4B). A strong phenotype similar to transformations induced by yki-RNAi or sd-RNAi was evident in ∼24% of hth-RNAi discs (Table S3); the other discs showed partial transformation. Similar results were obtained with a second, independent hth-RNAi line (Fig. S3C). Thus, we conclude that loss of Hth function results in a PE-to-DP transformation and development of an ectopic retina in the PE.

**Figure 5:**
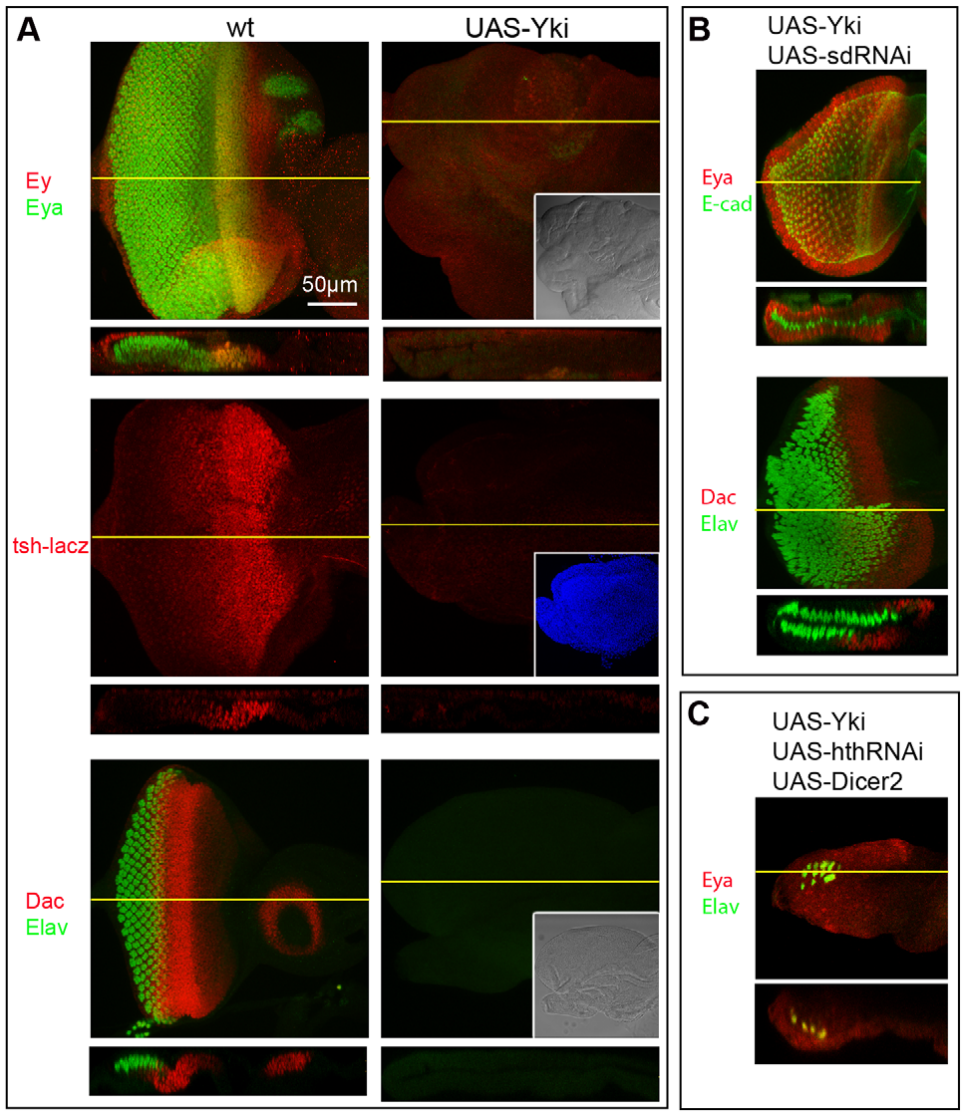
Suppression of retina formation by Yki over-expression depends on Sd and Hth. A) XY and ZX views of wt (left) and Yki over-expressing discs (right) stained for Ey (red) plus Eya (green) (top), *tsh-lacZ* (middle), and Dac (red) plus Elav (green) (bottom). Over-expression of Yki within the eye disc results in suppression of eye formation at the highest level (*tsh* and Ey) of the RD cascade. As shown in Fig. 4D, such discs express Hth throughout. These flies die as pharate adults that lack heads. Insets show the same discs under DIC (top and bottom panels), or with UAS-GFP expression (middle panel) to show discs' outlines. B) XY and ZX views of discs co-expressing Yki and sd-RNAi. Discs were stained for Eya (red) plus E-cadherin (green) (top) or Dac (red) plus Elav (green) (bottom); sd-RNAi not only restores eye formation in the DP layer of Yki-expressing discs, but also induces an ectopic retina in the PE (compare to wt and sd-RNAi disc in Fig. 1C). C) XY and ZX views of a disc co-expressing Yki, hth-RNAi, and dicer2 (to increase RNAi efficacy). Discs were stained for Elav (green) and Eya (red); hth-RNAi can restores eye development in Yki-expressing discs minimally and only in the DP cell layer (compare to wt in panel A and hth-RNAi disc in Fig. 4B).

To test whether Yki or Sd might mediate the effect of Hth as negative regulators of retina identity, Hth-GFP was over-expressed alone or together with sd-RNAi or yki-RNAi. As previously reported, discs with Hth-GFP over-expression were reduced in size and lacked the retina domain as shown by the lack of Eya and Elav expression (Fig. 5C; [24], [25]). Neither co-expression of sd-RNAi, nor co-expression of yki-RNAi, could restore eye development within the disc (Fig. 4C). Hence, Hth does not function upstream of Yki or Sd in promoting PE identity.

On the contrary, we found that Hth expression in the eye disc is controlled by Yki and Sd. Hth is expressed throughout the DP of Yki-expressing discs, whereas it acquires a DP-like pattern of expression (on in early progenitors, but off in the developing neuronal field) in the transformed PE of sd-RNAi discs (Fig. 4D).

In summary, Hth is indeed required for PE development and appears to function either in concert with or downstream of Yki and Sd in the establishment of PE-identity.

### Sd and Hth mediate Yki function

Since both Sd and Hth have been shown to bind Yki and provide functional specificity in some developmental contexts, we sought to investigate how Sd and Hth are related to Yki in the maintenance of PE identity and suppression of retinal fate. Therefore, we induced over-expression of Yki and then investigated how co-expression of sd-RNAi or hth-RNAi affected Yki function.

Since Yki activity is required to suppress retinogenesis in the PE cell layer, conversely, increasing Yki activity in the DP cell layer should interfere with its normal development. Over-expression of exogenous Yki throughout the eye disc resulted in greatly enlarged and disorganized discs with many folds in both epithelial layers. Although the cellular morphology of the Yki-expressing DP layer remained columnar, no neuronal development could be detected by Elav expression (Fig. 5A). This phenotype was also detected in Yki-overexpressing clones (MARCM method) though not with full penetrance (not shown). In addition, expression of the RDFs Ey, *tsh* (*tsh-lacz*), Eya and Dac was absent (Fig. 5A), indicating that formation and/or maintenance of an eye primordium was compromised. Therefore, high levels of Yki can suppress retina fate at the highest levels of the RD gene network.

To test whether Sd mediates the effect of Yki as a negative regulator of retina identity, Yki and sd-RNAi were expressed separately or together throughout the eye epithelium**.** Whereas Yki-expressing discs showed an abnormal DP layer, lacking expression of eye markers (Fig. 5A), co-expression of sd-RNAi led to formation of a double DP (Fig. 5B), very similar to what is observed in sd-RNAi-only discs (Figs. 1C, 2D, S3B). Thus, Yki cannot suppress retina identity without its partner Sd.

In similar experiments, Yki and hth-RNAi were expressed separately or together to test whether Hth, like Sd, is required for Yki to suppress retina identity. In this case, we found that although hth-RNAi could also restore retina markers in Yki-expressing discs, it could do so less strikingly than sd-RNAi. The expression of several RDFs was restored to different extents: Ey expression most robustly, Eya and Dac only weakly (Fig. S7). When Dicer 2 was also co-expressed, a stronger rescue of Eya and signs of neuronal development (Elav) were detected in some discs (4/20 discs; Fig. 5C). Contrary to what was observed in Yki sd-RNAi discs, expression of both markers was detected in only one cell layer (Fig. 5C). Incomplete knock-down of Hth might explain the less striking effect of hth-RNAi as compared to sd-RNAi, though staining with anti-Hth antibodies revealed a strong suppression of Hth expression (Fig. S2B; Table S3). Alternatively, Hth may mediate some, but not all, Yki functions in the PE and would thus work in a different capacity than Sd.

These findings, together with the previous evidence of *hth* regulation by Yki and Sd, suggest a model whereby Sd is absolutely required for Yki function in establishing PE identity, and Hth expression is initially induced or maintained by, and then contributes to, this Yki-Sd activity. Though this is consistent with Hth working as a downstream mediators of a Yki-Sd complex, since Hth has also been shown to complex with Yki [14], Hth could alternatively, or also, work as a component of a Yki-Sd-Hth complex.

### The Hippo pathway affects development of the eye primordium

The Yki transcription factor is a key component of the Hpo signaling pathway, and its partners (Sd and Hth) have been shown to contribute to the regulation of the pathway's proliferative and anti-apoptotic targets in the retina progenitor cells of the eye disc [12]–[15]. However, loss of proliferation or increased cell death do not appear to cause the type of tissue transformation we observed in the yki-RNAi or sd-RNAi backgrounds. In fact, neither a disc-wide (*ey-Flip; Act>IC>Gal4)* nor a PE-restricted *(pnr-Gal4)* loss of proliferation induces PE-to-DP transformations, nor can suppression of apoptosis in yki-RNAi- or sd-RNAi-expressing discs rescue the transformation phenotype (Fig. S8, and not shown). A possible scenario is that Yki and its partners mediate proliferation and survival in the context of the Hpo pathway, but control specification independently (through another genetic pathway). Alternatively, Hpo pathway components may regulate the activity of Yki complexes involved in all three processes, and differences in Yki activity levels and/or Yki partners may account for the different outputs.

To investigate whether the Hpo pathway was involved in the specification role played by these transcription factors, we manipulated the expression of pathway components that lie upstream of Yki, and then assessed development of the retina in the L3 disc. Since such components have a negative effect on Yki activity, we expected gain-of-function conditions of upstream factors to mimic loss-of- function *yki* phenotypes and, conversely, loss-of-function conditions to mimic gain-of-Yki-function.

**Figure 6:**
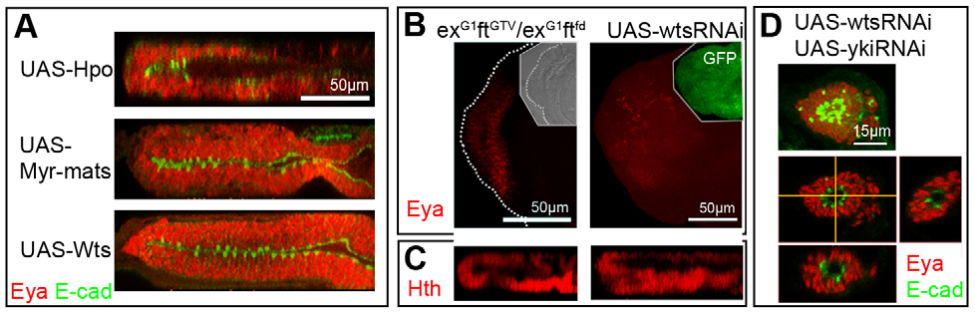
Changes in Hpo pathway activity can induce gain or loss of retina. A) ZX views of discs over-expressing negative regulators of Yki including Hpo (top), Wts (middle) and Myr-Mats (bottom). Ectopic retina development (red) occurs in the presumptive PE (compare to wt and transformed discs in Fig. 1C). B, C) XY (B) and ZX (C) views of discs double mutant for *ex ft* (left) or expressing wts-RNAi (right) stained for Eya (B) or Hth (C). Discs show reduced Eya and increased Hth expression. Discs do not form retinae based on Elav staining (not shown). Loss of Eya and increased Hth are also observed in Yki over-expressing discs (Fig. 5A). This is consistent with the increase in nuclear Yki activity expected in the absence of its upstream negative regulators. D) Co-expression of yki-RNAi in wts-RNAi expressing discs restores retina formation. Discs are similar to the most severe phenotype seen in yki-RNAi-only experiments, with no apparent PE. The eye-antennal discs are overall very small and the eye disc is shaped like a hollow ball of Eya-positive and Elav-positive cells. Top panel shows the XY view of entire eye discs or maximum projection of all confocal planes (max); bottom three panels show XY, ZX and ZY views of a single scanned, confocal plane.

For pathway gain-of-function, we over-expressed the wt forms of Hpo, Wts and a membrane-tethered (activated) form of Mats [26]-[28]. Interestingly, over-expression of these negative regulators of Yki mimicked the effect of yki-RNAi or sd-RNAi as reflected by the smaller disc size and PE-to-DP transformation (compare Fig. 6A to 1C). The RDF Eya was strongly expressed within 2 mirror-image, columnar epithelial layers (Fig. 6A). These results support a role for Yki in promoting PE formation and are consistent with a role for the Hpo pathway in regulating Yki activity in this process.

For pathway loss-of-function, we sought to compromise the upstream negative regulation of Yki. However, in this type of experiments, the endogenous level of Yki protein effectively limits the extent of hyper-activation that can be achieved by loss of a single upstream regulator (likely far below levels achieved with exogenous Yki). In order to achieve the highest possible level of endogenous Yki activation, we chose to investigate two specific genetic backgrounds. Since the negative regulation by upstream factors converges upon Yki through at least two separate branches [9], [29], we reasoned that a stronger activation of Yki might be achieved by simultaneously impairing both branches. This can be achieve in two ways: 1) by down-regulating Wts, the direct negative regulator of Yki upon which the branches converge, or 2) by knocking-down upstream components from each branch, as in the mutant combination of the upstream factors *fat (ft)* and *expanded (ex)* (*ft ex* double mutant) [30], [31]. We pursued both approaches and found that retina specification was indeed compromised in these genetic backgrounds. Expression of Eya in the DP layer was greatly reduced or nearly absent, whereas Hth expression was greatly expanded, in *ft ex* double mutant and wts-RNAi-expressing discs (Fig. 6B, C).

More importantly, down-regulation of Yki protein by expressing yki-RNAi in the wts-RNAi background led to a reversal of the block in retina development. Discs co-expressing yki-RNAi and wts-RNAi display a yki-RNAi-like double-retina phenotype (Fig. 6D). In fact, the wts-RNAi yki-RNAi discs are similar to the most severe phenotype seen in yki-RNAi experiments.

These findings are consistent with the known role of Wts as a negative regulator of Yki, and strongly support a model in which some level of down-regulation of Yki by upstream components of the pathway is required within the DP for the normal development of a retina primordium, despite the requirement for Yki in DP-cells' proliferation.

## Discussion

The process of regional specification in the eye-antennal disc of *Drosophila* involves a number of signaling pathways that regulate the expression of identity-defining transcriptional regulators, often referred to as selector factors. We show here that the Hippo signaling pathway and the transcription factors Yki, Sd, and Hth play critical roles in the regional specification of this disc. Specifically, all three factors are required for the establishment of the peripodial cell layer of the eye disc. Yki function is required in the PE to maintain tissue identity and appears to works in concert with Sd and Hth. Moreover, negative regulation of Yki through the Hippo/Warts genetic pathway is required in the DP to modulate Yki activity such that it promotes proliferation and survival of retina progenitor cells without interfering with their specification. The specification role of Yki and its partners in the eye epithelium is central to proper disc development, occurs in the early stages of regional specification within this disc and appears to be distinct from its more general role in proliferation and survival.

### Multiple functions of Yki and Sd in the eye disc

Yki and Sd have been shown to form a complex and regulate the anti-apoptotic gene *Diap1* in the wing and eye discs of *Drosophila* [12], [13]. In the eye, Yki is also strongly required for proliferation and survival, whereas Sd makes a lesser contribution to these processes [12]–[14].

Does Yki function in a complex with Sd in the context of regional specification within the eye disc and how? The evidence presented here shows that 1) the RNAi-induced knock-down of either gene induces essentially identical PE-to-DP transformations and that 2) sd-RNAi can suppress the gain-of-function, anti-retina effect of Yki over-expression. These findings, together with the biochemical evidence of protein-protein interactions [12], [13], [15], suggest that Yki and Sd may indeed control transcription together in a complex during PE specification.

Surprisingly, a comparison of loss-of-function analyses in mosaic discs versus RNAi-mediated disc-wide knock-downs has uncovered a discrepancy in the induced phenotypes. Specifically, loss-of-function clones of either *yki* or *sd* do not show signs of cell fate transformation. This is the case for traditional flip-FRT loss-of-function clones generated using mutant alleles [11], [12], [14] as well as for knock-down MARCM clones induced by RNAi expression (Fig. S4 this work and [13]). We do not believe that differences in reagents can explain this discrepancy, because the same yki-RNAi or sd-RNAi lines were used in our disc-wide versus clonal analyses. Hence, this discrepancy must depend on the two different loss-of-function approaches employed. The two approaches differ significantly in two ways: 1) the amount of mutant tissue induced, 2) and the timing of loss-of-gene function.

The first scenario would indicate that the Yki-Sd complex controls specification in a non-cell-autonomous fashion, such that, in mosaic discs, nearby wt cells would rescue the gene-silenced tissue. In this case, the simplest model is that Yki induces expression of a secreted factor that antagonizes retina identity. But other models are also possible. In support of this hypothesis, the secreted ligands of the Wingless (Wg), Egfr, and Jak/Stat signaling pathway (Wg, Vein and Unpaired respectively) have been shown to function downstream of Yki in different contexts providing evidence of non-cell-autonomous Hpo-Yki activity [32]-[37]. These pathways have been extensively studies in the fly eye and only Wg signaling, with its ability to suppress retina formation within the DP, fits the profile of likely candidate. However, down-regulation of Wg signaling, using its temperature sensitive allele Wg^ts^, does not result in the development of an ectopic retina within the PE (not shown). Thus, if a non-autonomous component is involved in the establishment of the PE, further analysis is needed to identify potential factors.

The second scenario would indicate a requirement for gene function at an earlier developmental stage than can be adequately probed through the mosaic approach. Generation of mosaic clones by FRT-mediated chromosomal-exchange is dependent on the start of cell proliferation late in L1. Hence, genotypically mutant cells begin to emerge in the late L1-early L2 stage and become phenotypically mutant somewhat later, once sufficient degradation of the gene products (mRNA/protein) has taken place. This characteristic renders clonal analysis problematic in studying phenotypes due to gene/protein activity prior to the mid to late L2 stage. On the contrary, in *ey-Flip Act>IC>Gal4 UAS-RNAi* discs, gene down-regulation begins in the embryonic stage because RNAi transgene expression initiates and becomes constitutive in the embryonic eye-antennal disc. Consistent with this scenario, we have identified the early to mid-late L2 interval as the critical period for Sd function in preventing the PE-to-DP transformation phenotype. Thus, traditional clonal analysis is indeed likely to be inadequate in assessing gene function requirements in this process. While this provides an explanation for our observations, it does not resolve the issue of a cell-autonomous versus non-cell-autonomous mechanisms. Further testing is needed and a method to eliminate gene function earlier, efficiently and in a proliferation-independent way will have to be developed to answer this question.

### Multiple functions of Hth in the eye disc

Thus far, two major functions of Hth have been uncovered in the DP cell layer: 1) Hth, together with Tsh, suppresses the expression of the late RD genes *eya* and *so*, thus slowing down the conversion of early eye progenitors (Ey, Tsh and Hth positive) into more mature (Ey, Tsh, Eya and So positive) eye precursor cells, and 2) Hth together with Tsh and Yki enhances proliferation by inducing expression of the proliferation-promoting microRNA *Bantam* [14], [17], [23]–[25], [38]. The combined effect of these two activities results in the generation of an abundant pool of eye progenitor cells. Protein-protein interactions among these factors have been documented *in vitro* and/or *in vivo*, leading to the proposal that two complexes (inclusive of Hth/Tsh and Hth/Yki/Tsh) may perform these major functions [14].

The data presented here uncover another critical function of Hth in the eye disc. In the PE cell layer, fated to give rise to portions of the head cuticle, Hth prevents conversion of this tissue into retina. As in the eye progenitors within the DP, this anti-retina activity of Hth involves suppression of late-RDFs (Eya, So, Dac). However, unlike its role in eye progenitors, it also entails down-regulation of the early-RDFs Ey and Tsh. Thus, Hth suppresses late-RDFs, but not early-RDFs, in early eye disc progenitors in conjunction with Tsh [24], whereas it suppresses retina formation at the level if the early-RDFs in the PE through a process that appears to involve Yki and Sd, but not Tsh (whose expression is normally absent from the PE). In agreement with this context dependent role of Hth, hth-RNAi reverses the anti-retina effect of Yki over-expression, at least in part, by relieving Yki suppression of the early-RDF Ey (Fig. S7), and, as shown by Bessa and Casares [20], expression of exogenous Tsh within the PE cell layer leads to the formation of an ectopic retina.

Lastly, we have shown that Hth expression is under the genetic, positive control of Yki and Sd, and that neither yki-RNAi nor sd-RNAi can suppress the gain-of-function activity of Hth. These findings, together with the reported association of the Hth and Yki proteins in S2 cells and on the regulatory region of *Bantam* [14], suggest a potentially more complex relationship between Hth and Yki in the development of the PE. The observation that Yki and Sd control Hth expression does not preclude the possibility that the Hth protein also functions in a complex with these factors. Indeed, a related example is offered by the RDF Eya, which first induces and then partners with the RDF Sine oculis to regulate retina development [3], [39], [40]. We propose therefore that Hth is one of the direct or indirect targets of a Yki/Sd complex in the PE. Thereafter, association with Hth may modify Yki/Sd activity eventually resulting in the transcriptional silencing of critical RDF genes, such as Tsh.

### Yki in proliferation versus specification

As summarized in the introduction, the Hippo/Yki signaling pathway is a general regulator of cell proliferation and survival in metazoans (recently reviewed in [29]). In a handful of cases, the Hippo pathway has been shown to regulate processes other than proliferation or cell death, such as the maintenance of the undifferentiated state of progenitor cell types in the neural tube and in the gut [41], [42] and, in two cases, aspects of neuronal differentiation. In one, the pathway controls the choice of opsin gene expressed in the R8 cells of the adult fly eye [43]; in the other, it is required for maintenance of the dendritic harbors of body-wall sensory neurons in the *Drosophila* larva [44]. While transcriptional regulation by Yki is thought to play a role in these non-proliferation-related processes, it is difficult to draw parallels between these cases and what we see in the eye disc.

Two more recent reports implicate Yki in processes related to tissue specification. In one case, a transient, expanding burst of Yki activity within rows of cells at the border of an already established wing field results in a marginal expansion of the wing primordium, ultimately ensuring proper wing size [45]. In a second example, the YAP/Yki and TEAD4/Sd proteins specify the trophoectoderm as distinct from the inner cell mass (ICM) in mammalian embryos [46]. It is thought that the circumferential cell-cell contacts experienced by ICM cells triggers Hpo activity and cytoplasmic retention of YAP, whereas the less extensive cell-cell contacts experienced by outer cells do not, thus allowing nuclear accumulation of YAP [46], [47].

Based on similarities with the latter example, it is tempting to hypothesize that less extensive cell-cell contacts in the squamous cell layer, than in the columnar one, would promote a more efficient nuclear localization of Yki in the PE, than in the DP cells. At moderate activity levels in the DP, Yki would then promote proliferation and survival without interfering with retina specification, whereas at higher activity levels in the PE, Yki would be able to not only promote cell proliferation and survival but also PE-identity by suppressing retina formation. This scenario would be consistent with the observed need for down-regulation of Yki activity by Warts in the DP (Fig. 6) and the stronger expression of *Diap1-2-lacZ* in the PE (Fig. 3B). However, the observation that the two cell layers differ significantly in the availability of factors found in Yki-based complexes [14], [20] suggests that ‘Yki-complex composition’ plays a critical role in the PE versus DP/retina distinction in the developing fly eye.

**Figure 7:**
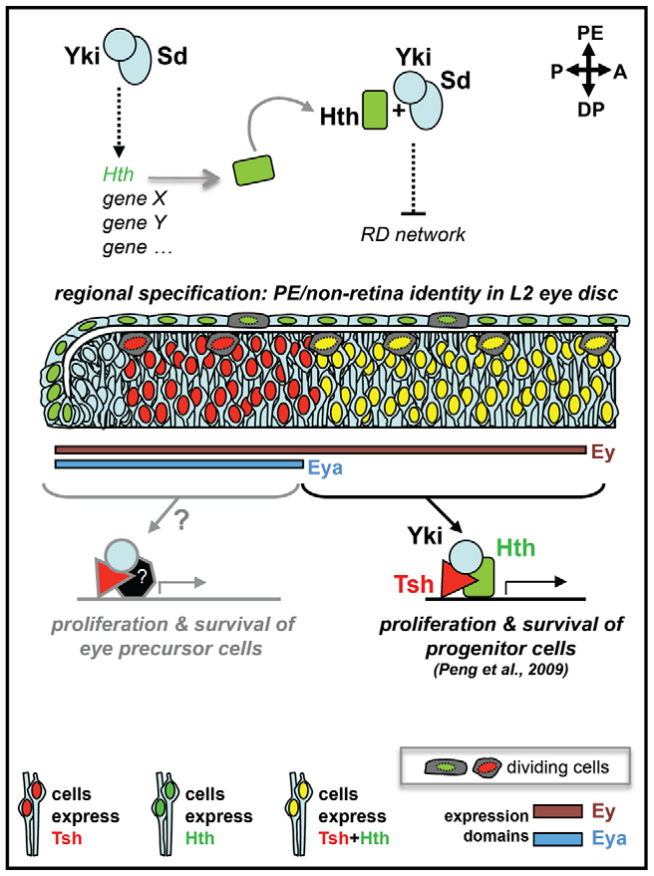
Yki, Sd and Hth functions in different regions of the L2 eye disc. In this model, different combinations of co-factors associate with Yki to bring about different outcomes in distinct regions of the L2 eye disc epithelium. A Yki/Hth/Tsh complex would ensures the maintenance and expansion of the retina progenitors pool in the DP cell layer by promoting proliferation and suppressing apoptosis; whereas, in the PE, Hth is first induced by, and then would work in concert with, Yki and Sd to control regional specification by suppressing retina identity and promoting development of PE derivatives (this work). The induction of Hth by Yki/Sd could be direct or indirect (dashed arrow), whereas the negative regulation of one or more genes of the RD network would have to be, at least in part, indirect based on the non-cell-autonomous features of mutant clones. Whether Yki, together with Tsh and/or other partners, controls proliferation and survival of the more developmentally advanced ‘Eya-positive + Hth-negative’ retina progenitors (named here “eye precursor” cells) is likely, based on the small size of yki-RNAi expressing clones from this region of the disc, but requires further testing. Though not shown in this diagram for simplicity, Ey is also expressed throughout the PE and Eya is expressed in a restricted posterior-lateral portion of the PE, in the L2 eye disc; as shown, Tsh is detected only in the DP cell layer.

Yki association with specific co-factors would, therefore, modify the output of the Hpo-Yki pathway to bring about different outcomes in distinct regions of the eye epithelium. A Yki/Hth/Tsh complex would ensure maintenance and expansion of the retina progenitors pool [14], whereas a Yki/Sd, and possibly Hth, complex would contribute to regional specification within the eye disc by ensuring formation of PE-derived head structures (this work) (Fig. 7). That Yki/YAP-based complexes can include a variety of different co-factors is supported by other recent examples, including the above mentioned role of Yki, Hth and Tsh in promoting eye progenitors' proliferation in the fly [14], and its interaction with IRS1 [48] to promote proliferation of neural precursors or with Smad1 [49] to enhance BMP-mediated suppression of neuronal differentiation of embryonic stem cells in the mouse.

In the L2 eye disc, the Sd protein is believed to be available throughout, but does not appear to contribute greatly to Yki-induced proliferation in either cell layer (Fig. S4; [14]). On the contrary, in the PE, it behaves as a critical co-factor in the control of tissue identity. Unlike Sd, Tsh expression has been shown to be restricted specifically to the DP cell layer [20]. Thus, Yki must perform its PE-promoting tasks in transcriptional complexes that do not include Tsh. Interestingly, misexpression of Tsh within the PE has been shown to induce retina development in this tissue [20]. Whether Tsh does so, at least in part, by diverting Yki activity away from ‘anti-retina-fate’ functions remains to be determined. Hth, also present broadly in both cells layer at the L2 stage, would play a more general role contributing to Yki's function in both proliferation and tissue specification.

One possible scenario is that the tissue specification functions of Yki in the PE are critically dependent on its association with Sd, and that the availability of Tsh specifically in the DP interferes with the ability of Sd and Yki to associate or work together on fate-determining tasks, thereby preventing any interference with retina formation.

As shown by the paucity of examples, we are still in the early days of deciphering how Yki and its partners regulate cell fate. Nonetheless, this role is apparently separate from its contribution to cell proliferation and survival, and likely involves a number of distinct molecular mechanisms including co-factor specificity and differing levels of Yki activity [14,45,46, this work].

## Materials and Methods

### *Drosophila* stocks

For targeted expression of RNAi or protein, we used the drivers *pnr-Gal4*, or *ey-Gal4* or the flip-out UAS/Gal4 system [50], *ey-Flip* with *Actin5C>y^+^>Gal4* or *Actin5C>CD2>Gal4*, referred to as *Act>Interruption-Cassette>Gal4* or *Act>IC>Gal4* [3], [51], [52]. TRiP-Harvard Medical School (NIH/NIGMS R01-GM084947) transgenic RNAi stocks: *UAS-sdRNAi* (JF02514), *UAS-ykiRNAi* (HMS00041), *UAS-hthRNAi* (HMS01112); *UAS-CycERNAi* (JF02473), *UAS-mycRNAi* (JF01761), and *UAS-NotchRNAi* (JF01891) [53], [54]. VDRC RNAi stocks: *UAS-hthRNAi* (12763) [55], *UAS-wtsRNAi* (106174) [37]. Jiang's RNAis: *UAS-sdRNAi^N^*, *UAS-sdRNAi^ N+C^*, *UAS-ykiRNAi^N^*, *UAS-ykiRNAi^C^* [13]. Other lines: *ey-Gal4* [56], *UAS-hpo* [26], *UAS-yki* [11], *UAS-Myc-wts* [27], *UAS-Myr-mats* [28], *UAS-hthGFP* [57], *UAS-dicer2* (VDRC # v60009), *UAS-P35*, *UAS-GFP*, *dpp-lacz*, *tsh-lacz* (*tsh^1^*), *tub-Gal80^ts^* (Bloomington), D*iap1-2-lacZ* [12], *ex^G1^ ft^GTV^/ Cyo GFP* and *ex^G1^ ft^fd^/ Cyo* [30].

### Genetics crosses

*ey-Flip Act>IC>Gal4* drivers were used in all experiments unless explicitly stated. See Table S1 for detailed genotypes and temperatures. The yki-RNAi-expressing clones were generated by the *ey-Flip* MARCM method [58]. Gal80^ts^ temperature shift experiments: Sd protein expression was altered by shifting temperature between 18°C (Sd expressed; Gal80**^ON^** → RNAi**^OFF^**) and 29°C (Sd not-expressed; Gal80**^OFF^** → RNAi**^ON^**). Control progeny (e.g. maintained at 18°C throughout) developed into normal adults. Development proceeds more than twice as fast at 29°C than at 18°C. Embryos were collected from cages for 1 hr at room temperature, plates were then placed at 18°C or 29°C as needed until time of shift. Dissections were carried out at the L2 and L3 stage as needed, head phenotypes were scored at the adult stage. Adult head phenotypes (I–V) that correlate with varying degrees of PE-to-DP transformation in the developing disc were identified by combining the analysis at adult and larval stages for selected time points in the shift from 18°C to 29°C. At early-mid L2 (96 hour AEL at 18°C) induced high percentage of adults with protruding eyes (III), severely deformed heads (IV) or headless adults with eyes fused to the thorax (V) (Figs. 3C, S6C). Analysis of this sample at the late L3 stage showed eye discs with complete or nearly complete PE-to-DP transformation (Fig. S6). On the contrary, shifts of developing larvae at 144 h AEL (early L3 stage) induced phenotypes not-associated with PE-to-DP transformation (I, II) (Figs. 3C, S6C). The shift at 120 h AEL (mid-late L2 stage) induced various phenotypes from II to IV (Figs. 3C, S6C), and the discs also showed complete, partial, or no transformation (Fig. S6).

### Immunohistology

Antibody stainings of discs (≥12 scored, unless explicitely stated) were as per standard protocol. Primary antibodies - DSHB (http://dshb.biology.uiowa.edu/): mouse anti-Eya (1∶100), mouse anti-Dac (1∶200), mouse anti-DLG (1∶100), rat anti-Elav (1∶20), rat anti-E-cad (1∶20)- other sources: rabbit anti-β-galactosidase (1∶1000, Cappel), goat anti-Hth (1∶200, Santa Cruz), rabbit anti-Ey (1∶2000) [59], rabbit anti-Yki [31]. Cy2, Cy3, or Cy5 secondary antibodies conjugated (Jackson Immuno Research Laboratories) were used at 1∶200. Images were obtained with a Leica DM5500Q confocal system and processed with Adobe-Photoshop.

## Supporting Information

Figure S1 **Disc development, expression of molecular markers and Gal4.** A) Detailed schematic of developing L3 eye disc and expression patterns of molecular markers used to identify eye disc cell types in this work. B) XY and ZX views of GFP expression in developing eye discs expressing UAS-GFP under the control of the “*ey-Flip; Actin>IC>Gal4*” drivers. The *ey-Flip; Actin>IC>Gal4* combination drives expression broadly within the eye-antennal disc; all cells of the eye disc and most but not all cells of the antenna express the UAS-GFP transgene. The UAS-RNAi transgenes were co-expressed with *UAS-dicer 2* (to enhance the efficiency of the RNAi) and *UAS-GFP* (to mark the expressing tissue) under the control of *ey-Flip; Actin>IC>Gal4* drivers in all experiments unless otherwise stated (exact genotypes of all discs shown in regular and supplemental figures are listed in Table S1).
(PDF)

Figure S2 **yki-RNAi and hth-RNAi efficiently down-regulate the expression of Yki or Hth respectively.** A) L3 disc containing clones of yki-RNAi expressing cells (marked by GFP, green) stained with an anti-Yki Ab; XY shows DP cell layer. The Yki protein is efficiently down-regulated in yki-RNAi expressing cells, but continues to be robustly expressed in both cell layers in other regions of the disc. B) L3 discs stained with anti-Hth Ab. Hth is strongly expressed in the wt (left) or in Yki over-expressing (middle) discs, but cannot be detected in the presence of hthRNAi.
(TIF)

Figure S3 **Multiple RNAi lines induce consistent mutant phenotypes.** A, B) transgenic yki-RNAi (A) and sd-RNAi (B) lines from the Jiang's lab also induce strong PE-DP transformation phenotypes when expressed in the eye disc, as does the TRiP hth-RNAi line HMS01112 as well (C). These and the lines shown in Fig. 1C are directed against different regions of their respective mRNA targets (see Table S2). Hence, their effect is due to the down-regulation of the intended mRNA targets. In addition, another Gal4 line, *ey-Gal4*, with a similar expression also induced the transformation phenotype (not shown), indicating the effect is not restricted to the binary Flip-out Gal4 driver.
(TIF)

Figure S4 **Loss of** ***sd*** **or** ***yki*** **function in clones does not induce ectopic retina formation in the PE.** ZX (top) and XY (bottom) views of L3 discs with clones expressing GFP (left), GFP + sd-RNAi (middle), or GFP + yki-RNAi (right). No PE-to-DP/retina transformation was observed in any yki-RNAi- or sd-RNAi-expressing clones; yki-RNAi-expressing clones were consistently smaller than wt clones, reflecting the dramatic effect of loss of Yki on proliferation; sd-RNAi-expressing clones were somewhat smaller than wt but significantly larger than yki-RNAi clones. Ten wt (3 discs), 32 sdRNAi (4 discs), and 40 ykiRNAi (6 discs) clones all within the PE cell layer were individually scored for the presence of ectopic of Eya expression in XY, ZX and ZY views. None was detected.
(TIF)

Figure S5 **Photoreceptor neurons axonal projections in wt and transformed discs.** XY views of wt and sd-RNAi discs stained with 22C10 to highlight axonal projections. The axons emanating from the transformed-PE display an overall organization remarkably similar to the axonal projections from the normal DP layer. Arrows mark the nerve of the Bolwig's or larval eye. This axonal bundle extends from neuronal cell bodies located in the larval head, in close contact with the PE cell layer of the eye disc (basal side), and into the brain along the optic stalk (see also 22C10 panels in Fig. 2C for ZX views from another disc).
(TIF)

Figure S6 **Temperature shift experiment and correlation of adult and disc phenotypes induced by sd-RNAi expression.** A–D) Range of phenotypes seen in flies and pharate adults (A) in temperature shift experiments, corresponding L3 disc abnormalities (B), and penetrance as percentage of flies displaying a specific phenotype (C, D). Discs with signs of PE-to-DP/retina transformation result in type III, IV or V adult fly phenotypes, whereas discs that show no evidence of transformation produce type I (normal) or type II flies. Total number of flies scored in (C) and (D) is marked above each time point. Adult head phenotypes (III–V) that correlate with varying degrees of PE-to-DP transformation in the developing disc were identified by analysis at adult and larval stages for selected time points. The shift from 18°C to 29°C at early-mid L2 (96 hour AEL at 18°C) induced mostly adults with protruding eyes (III), severely deformed heads (IV) or headless adults with eyes fused to the thorax (V) (panel C). Analysis of this sample at the late L3 stage shows eye discs with complete or nearly complete transformations (panel B, 96 hr top). On the contrary, shifts at 144 h AEL (early L3 stage, panel B, bottom) induced only phenotypes not-associated with the transformation of the PE (I, II) (panel C). The shift at 120 h AEL (mid-late L2 stage, panel B, middle) induced various phenotypes from II to IV (panel C), and the discs also show complete, partial, or no transformations (panel B). C, D) Using this approach, we identified the time between the L1/L2 molt and mid-late L2 as the phenocritical period for Sd gene function. Panel C shows that transformation-related phenotypes (III, IV, V) are seen at high penetrance whenever gene silencing (shift up) began prior and up to mid L2 (108 hr). At mid-late (120 hr) and late L2 (132 hr), a drastic drop in transformation occurs, first down to ∼50% and then to ∼15% (See Fig. 3D); later time points show no transformation-related phenotypes. Thus, inactivation of endogenous Sd function any time between embryogenesis and 120 hr (18°C), but not later, compromises tissue identity in the PE indicating that protein activity is required until ∼mid-late L2 to promote PE identity. Conversely, panel D shows that restoration of Sd function (by shift-down) prior to mid-late L1 (30 hr, 29°C) resulted in minimal disruption of PE formation (non-transformation phenotypes in ≥80%); whereas Sd restoration at the L1/L2 molt (36 hr, 29°) or thereafter (in L2 and L3) was insufficient to ensure proper PE development (the penetrance of the transformation phenotypes increase from ∼50% in early L2 to 100% by late L2). Thus, we conclude that Sd function becomes necessary to promote PE identity very late in L1 or early in L2. This result is consistent with the emergence of an Eya-positive ectopic retina domain during the L2 stage shown in Fig. 3A.
(TIF)

Figure S7 **Co-expression of the hth-RNAi only partially reverses the loss of eye primordium induced by Yki over-expression.** XY and ZX views of Yki and hth-RNAi expressing discs. Discs were stained for Ey, Eya or Dac (red) as marked on each panel. Insets and ZX-projections in Eya and Dac panels show digitally-enhanced expression of Dac and Eya for clarity.
(TIF)

Figure S8 **Changes in cell proliferation or cell death do not alter regional identity within the disc.** A) ZX views of RNAi-expressing discs stained for Eya (red) and E-cad (green). RNAi knock-down of the G1>S transition CycE, the growth regulator Myc, or Notch, a critical regulator of proliferation in the eye primordium, produced discs that were overall smaller in size than sd-RNAi expressing discs. Nonetheless, overall disc morphology was undisturbed with a thin, squamous PE cell layer devoid of any signs of retina formation and an Eya-positive, columnar DP cell layer. B) Suppression of apoptosis by co-expression of the baculovirus anti-apoptosis factor P35 does not suppress the transformation phenotype of yki-RNAi discs. Except for a decrease in activated-Caspase3 positive cells, yki-RNAi (top) and yki-RNAi+P35 (bottom) discs are nearly indistinguishable.
(TIF)

Table S1 **List of genotypes analyzed and related figures.**
(DOC)

Table S2 **RNAi lines used in this work.**
(DOC)

Table S3 **Relative strength of gene silencing.**
(DOC)
