# Supplementary figures and images for: Yki/YAP, Sd/TEAD and Hth/MEIS Control Tissue Specification in the Drosophila Eye Disc Epithelium
Source: PLoS One. 2011 Jul 19;6(7):e22278. doi: 10.1371/journal.pone.0022278 (PMC3139632; doi:10.1371/journal.pone.0022278)

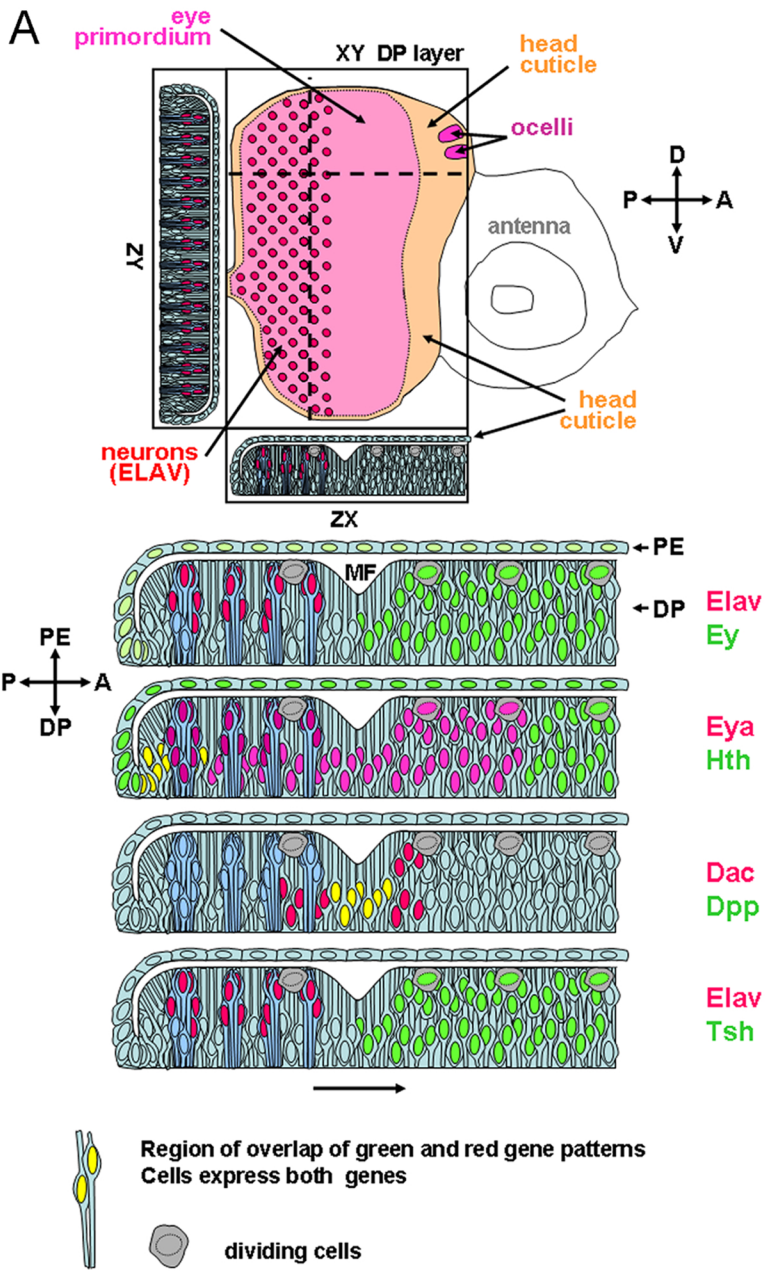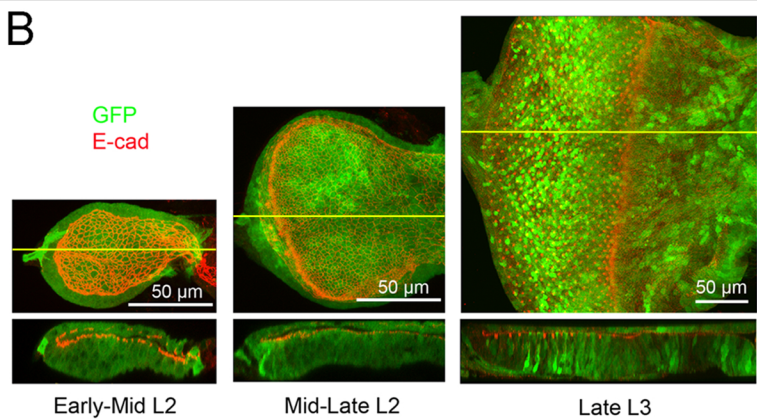

Supplement: Figure S1 — Disc development, expression of molecular markers and Gal4. A) Detailed schematic of developing L3 eye disc and expression patterns of molecular markers used to identify eye disc cell types in this work. B) XY and ZX views of GFP expression in developing eye discs expressing UAS-GFP under the control of the “ey-Flip; Actin>IC>Gal4” drivers. The ey-Flip; Actin>IC>Gal4 combination drives expression broadly within the eye-antennal disc; all cells of the eye disc and most but not all cells of the antenna express the UAS-GFP transgene. The UAS-RNAi transgenes were co-expressed with UAS-dicer 2 (to enhance the efficiency of the RNAi) and UAS-GFP (to mark the expressing tissue) under the control of ey-Flip; Actin>IC>Gal4 drivers in all experiments unless otherwise stated (exact genotypes of all discs shown in regular and supplemental figures are listed in Table S1). (PDF) [file pone.0022278.s001.pdf]

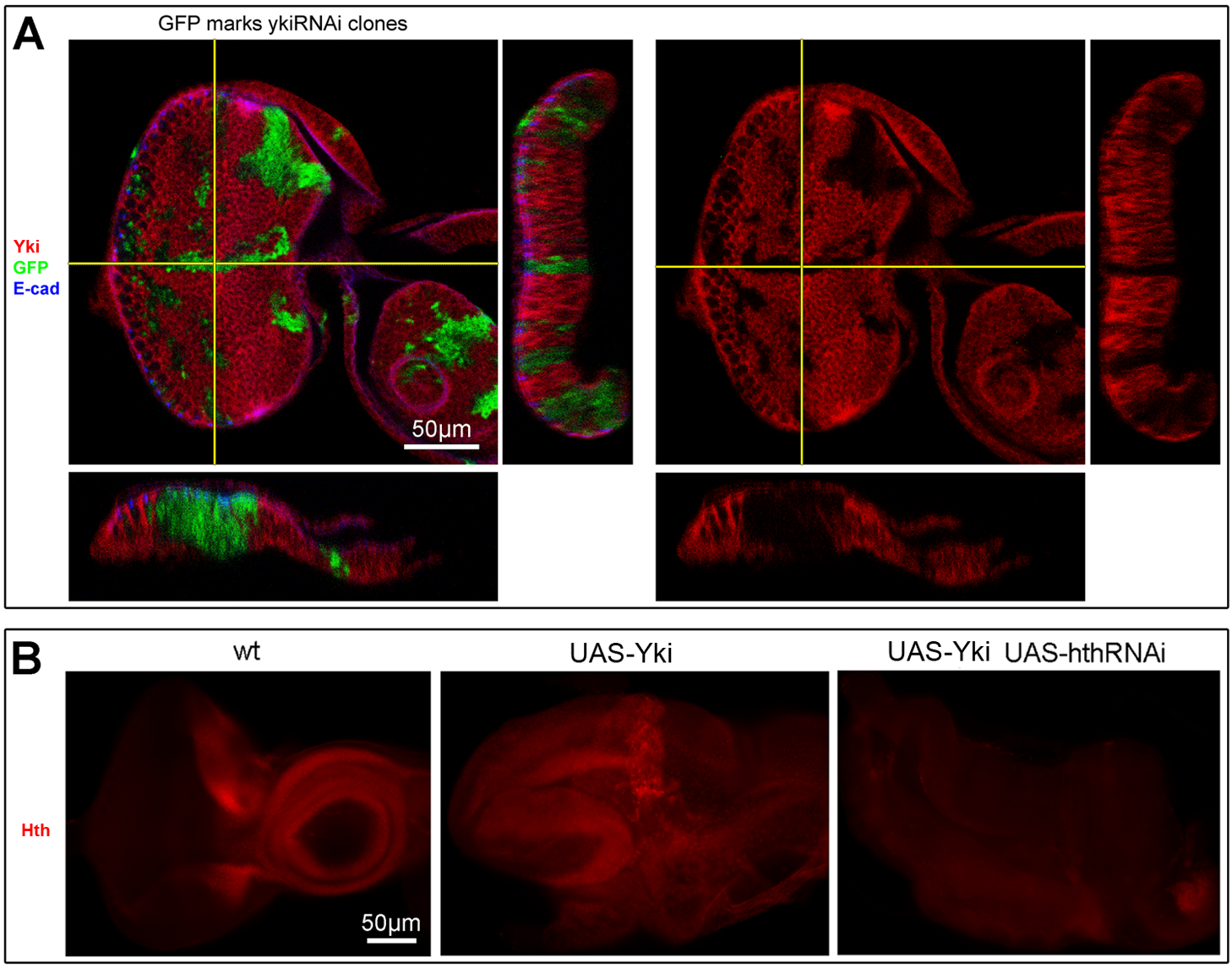

Supplement: Figure S2 — yki-RNAi and hth-RNAi efficiently down-regulate the expression of Yki or Hth respectively. A) L3 disc containing clones of yki-RNAi expressing cells (marked by GFP, green) stained with an anti-Yki Ab; XY shows DP cell layer. The Yki protein is efficiently down-regulated in yki-RNAi expressing cells, but continues to be robustly expressed in both cell layers in other regions of the disc. B) L3 discs stained with anti-Hth Ab. Hth is strongly expressed in the wt (left) or in Yki over-expressing (middle) discs, but cannot be detected in the presence of hthRNAi. (TIF) [file pone.0022278.s002.tif]

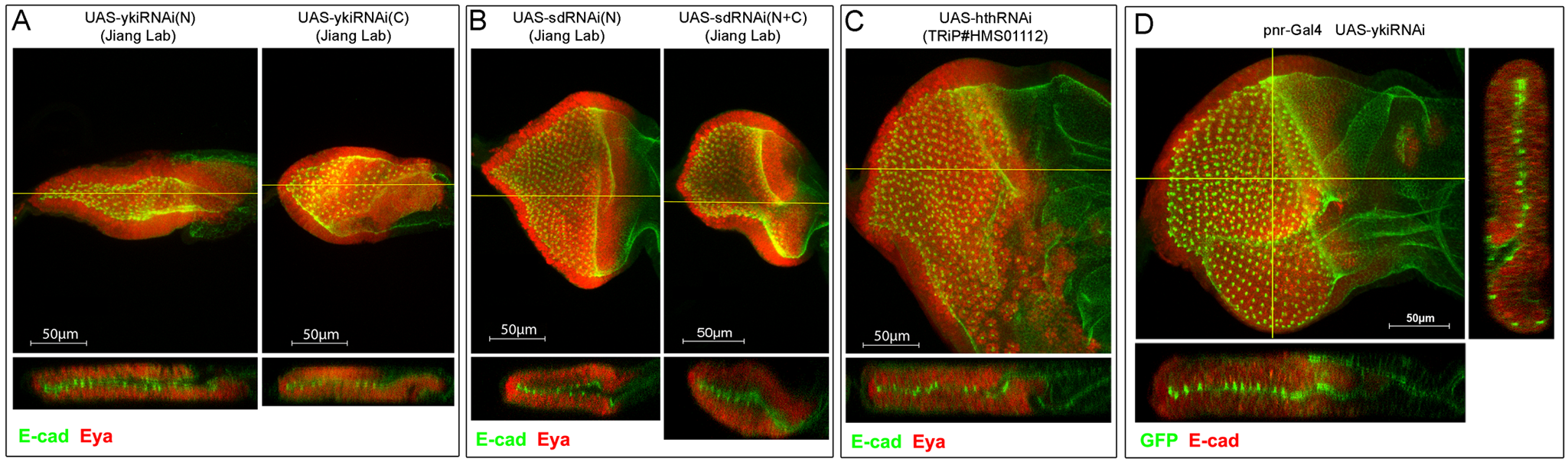

Supplement: Figure S3 — Multiple RNAi lines induce consistent mutant phenotypes. A, B) transgenic yki-RNAi (A) and sd-RNAi (B) lines from the Jiang's lab also induce strong PE-DP transformation phenotypes when expressed in the eye disc, as does the TRiP hth-RNAi line HMS01112 as well (C). These and the lines shown in Fig. 1C are directed against different regions of their respective mRNA targets (see Table S2). Hence, their effect is due to the down-regulation of the intended mRNA targets. In addition, another Gal4 line, ey-Gal4, with a similar expression also induced the transformation phenotype (not shown), indicating the effect is not restricted to the binary Flip-out Gal4 driver. (TIF) [file pone.0022278.s003.tif]

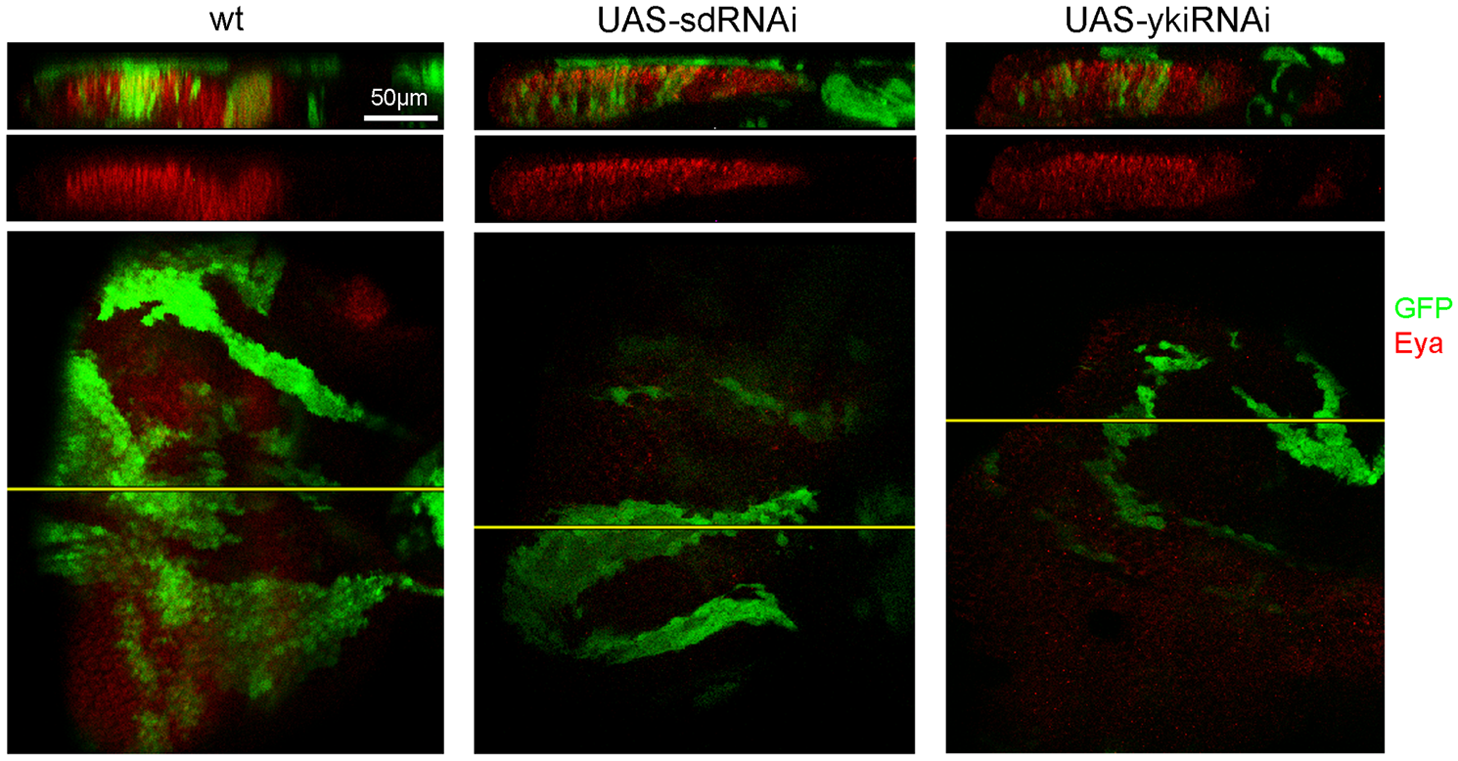

Supplement: Figure S4 — Loss of sd or yki function in clones does not induce ectopic retina formation in the PE. ZX (top) and XY (bottom) views of L3 discs with clones expressing GFP (left), GFP + sd-RNAi (middle), or GFP + yki-RNAi (right). No PE-to-DP/retina transformation was observed in any yki-RNAi- or sd-RNAi-expressing clones; yki-RNAi-expressing clones were consistently smaller than wt clones, reflecting the dramatic effect of loss of Yki on proliferation; sd-RNAi-expressing clones were somewhat smaller than wt but significantly larger than yki-RNAi clones. Ten wt (3 discs), 32 sdRNAi (4 discs), and 40 ykiRNAi (6 discs) clones all within the PE cell layer were individually scored for the presence of ectopic of Eya expression in XY, ZX and ZY views. None was detected. (TIF) [file pone.0022278.s004.tif]

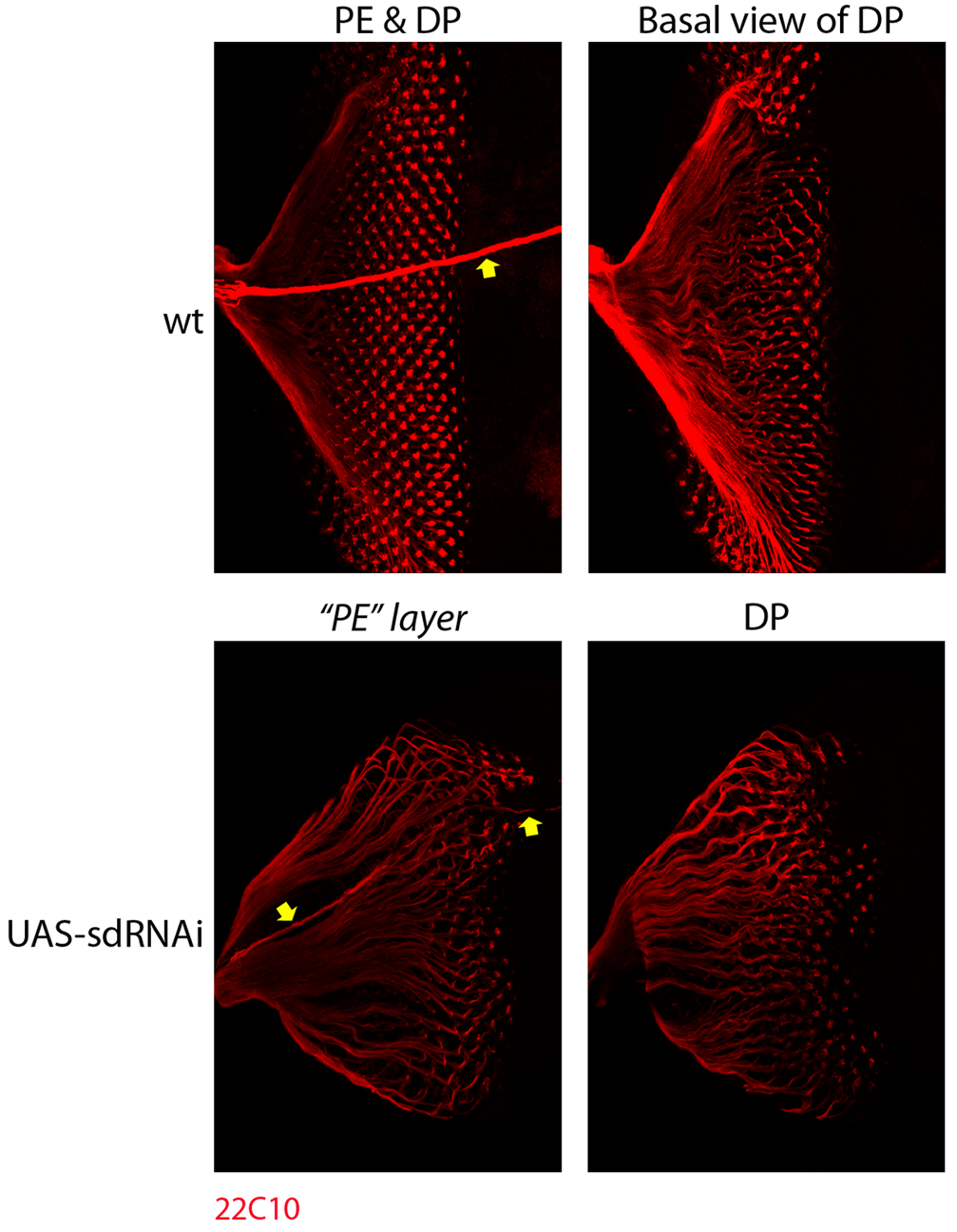

Supplement: Figure S5 — Photoreceptor neurons axonal projections in wt and transformed discs. XY views of wt and sd-RNAi discs stained with 22C10 to highlight axonal projections. The axons emanating from the transformed-PE display an overall organization remarkably similar to the axonal projections from the normal DP layer. Arrows mark the nerve of the Bolwig's or larval eye. This axonal bundle extends from neuronal cell bodies located in the larval head, in close contact with the PE cell layer of the eye disc (basal side), and into the brain along the optic stalk (see also 22C10 panels in Fig. 2C for ZX views from another disc). (TIF) [file pone.0022278.s005.tif]

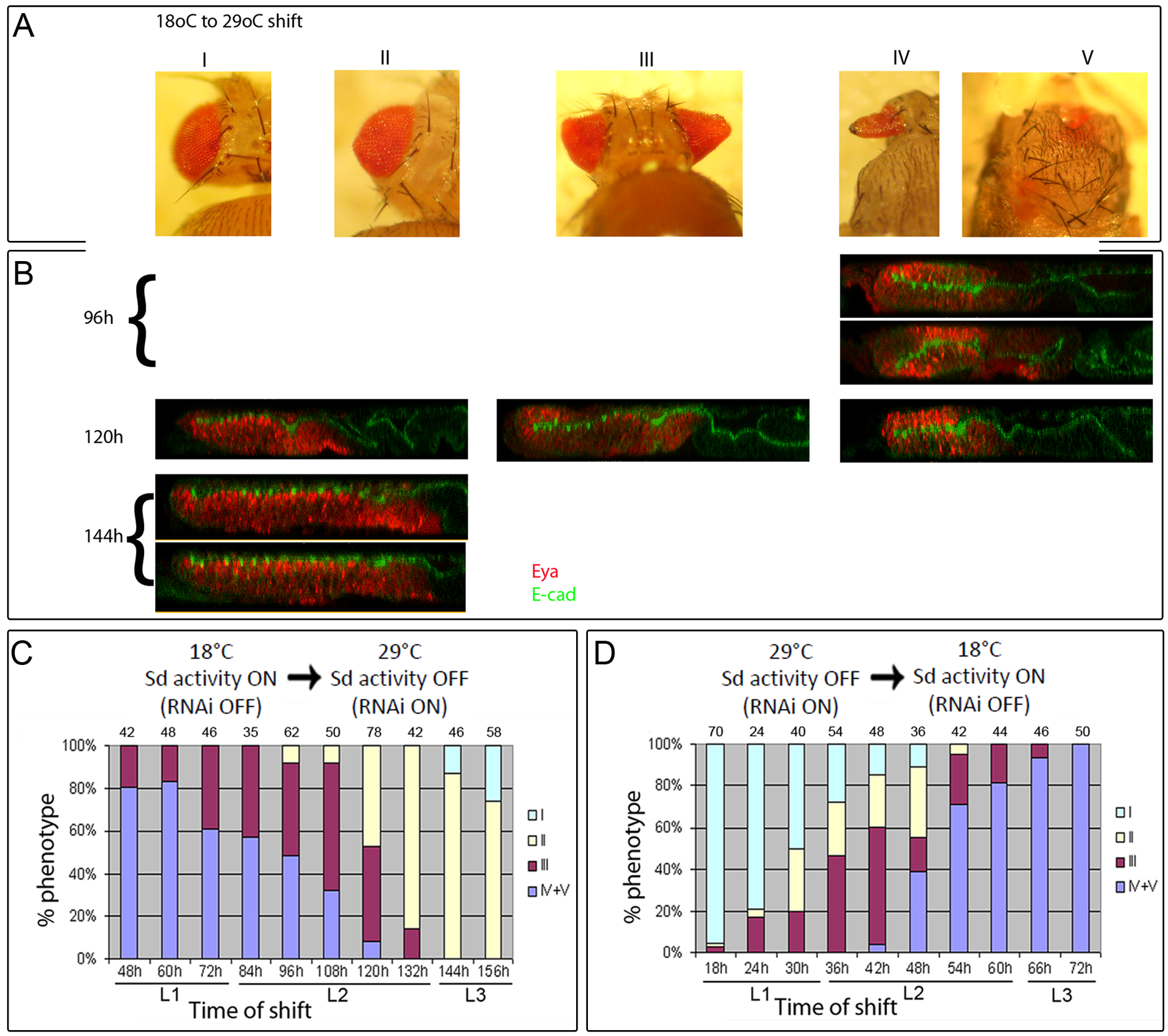

Supplement: Figure S6 — Temperature shift experiment and correlation of adult and disc phenotypes induced by sd-RNAi expression. A–D) Range of phenotypes seen in flies and pharate adults (A) in temperature shift experiments, corresponding L3 disc abnormalities (B), and penetrance as percentage of flies displaying a specific phenotype (C, D). Discs with signs of PE-to-DP/retina transformation result in type III, IV or V adult fly phenotypes, whereas discs that show no evidence of transformation produce type I (normal) or type II flies. Total number of flies scored in (C) and (D) is marked above each time point. Adult head phenotypes (III–V) that correlate with varying degrees of PE-to-DP transformation in the developing disc were identified by analysis at adult and larval stages for selected time points. The shift from 18°C to 29°C at early-mid L2 (96 hour AEL at 18°C) induced mostly adults with protruding eyes (III), severely deformed heads (IV) or headless adults with eyes fused to the thorax (V) (panel C). Analysis of this sample at the late L3 stage shows eye discs with complete or nearly complete transformations (panel B, 96 hr top). On the contrary, shifts at 144 h AEL (early L3 stage, panel B, bottom) induced only phenotypes not-associated with the transformation of the PE (I, II) (panel C). The shift at 120 h AEL (mid-late L2 stage, panel B, middle) induced various phenotypes from II to IV (panel C), and the discs also show complete, partial, or no transformations (panel B). C, D) Using this approach, we identified the time between the L1/L2 molt and mid-late L2 as the phenocritical period for Sd gene function. Panel C shows that transformation-related phenotypes (III, IV, V) are seen at high penetrance whenever gene silencing (shift up) began prior and up to mid L2 (108 hr). At mid-late (120 hr) and late L2 (132 hr), a drastic drop in transformation occurs, first down to ∼50% and then to ∼15% (See Fig. 3D); later time points show no transformation-related phenotypes. Thus, inactiva [file pone.0022278.s006.tif]

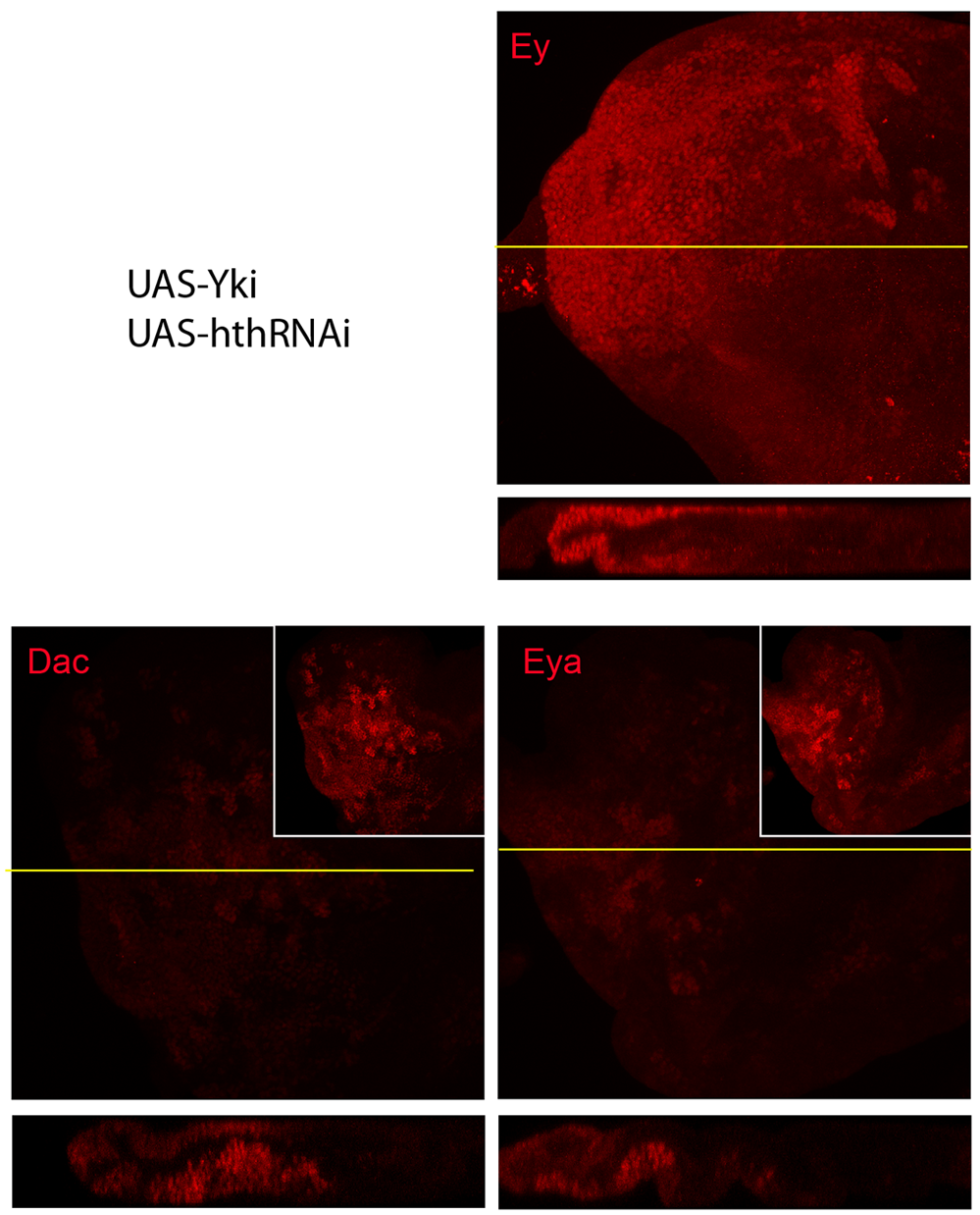

Supplement: Figure S7 — Co-expression of the hth-RNAi only partially reverses the loss of eye primordium induced by Yki over-expression. XY and ZX views of Yki and hth-RNAi expressing discs. Discs were stained for Ey, Eya or Dac (red) as marked on each panel. Insets and ZX-projections in Eya and Dac panels show digitally-enhanced expression of Dac and Eya for clarity. (TIF) [file pone.0022278.s007.tif]

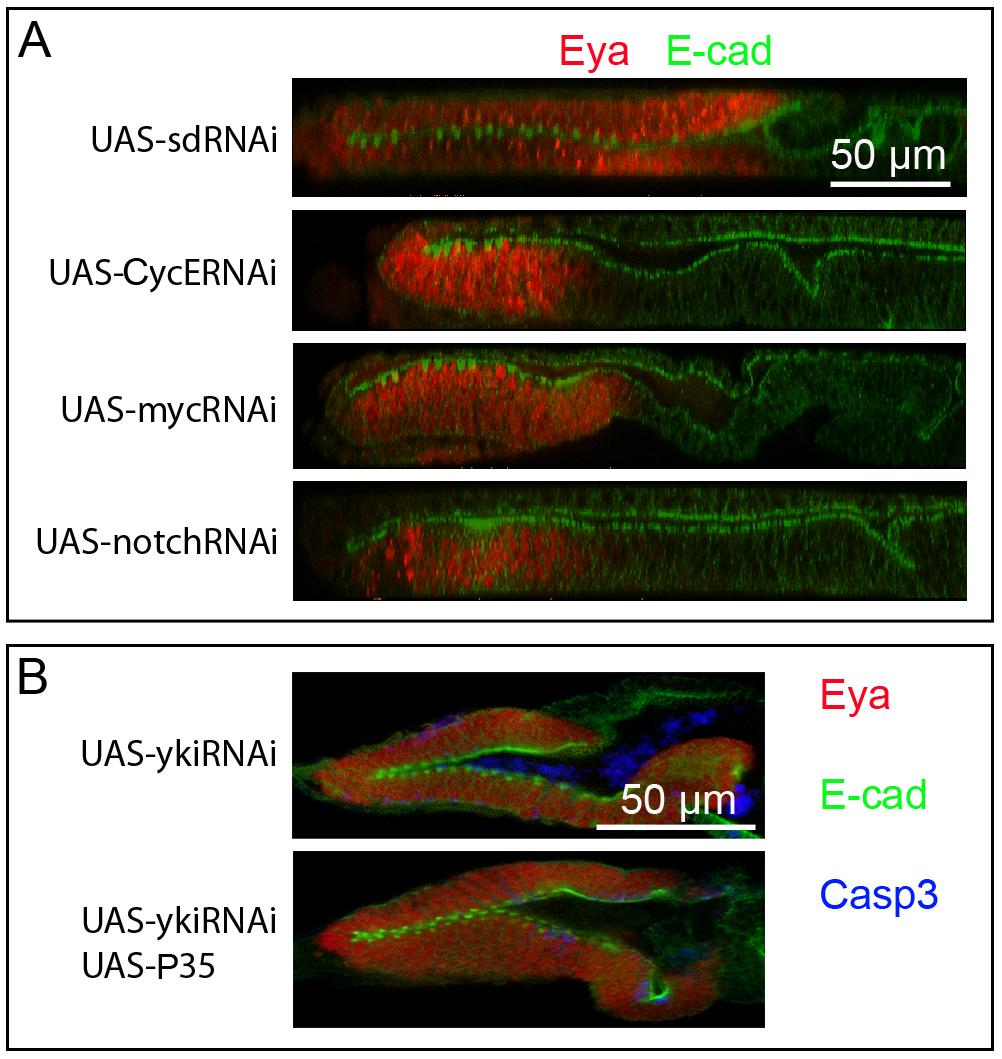

Supplement: Figure S8 — Changes in cell proliferation or cell death do not alter regional identity within the disc. A) ZX views of RNAi-expressing discs stained for Eya (red) and E-cad (green). RNAi knock-down of the G1>S transition CycE, the growth regulator Myc, or Notch, a critical regulator of proliferation in the eye primordium, produced discs that were overall smaller in size than sd-RNAi expressing discs. Nonetheless, overall disc morphology was undisturbed with a thin, squamous PE cell layer devoid of any signs of retina formation and an Eya-positive, columnar DP cell layer. B) Suppression of apoptosis by co-expression of the baculovirus anti-apoptosis factor P35 does not suppress the transformation phenotype of yki-RNAi discs. Except for a decrease in activated-Caspase3 positive cells, yki-RNAi (top) and yki-RNAi+P35 (bottom) discs are nearly indistinguishable. (TIF) [file pone.0022278.s008.tif]
