# Supplementary material for: Yki/YAP, Sd/TEAD and Hth/MEIS Control Tissue Specification in the Drosophila Eye Disc Epithelium
Source: PLoS One. 2011 Jul 19;6(7):e22278. doi: 10.1371/journal.pone.0022278 (PMC3139632; doi:10.1371/journal.pone.0022278)
Supplement: Table S1 — List of genotypes analyzed and related figures. (DOC) [file pone.0022278.s009.doc]

**Supplemental Table S1: list of genotypes analyzed**

| **Figure #** | **Genotype^(1)^** | **Temperature** |
| --- | --- | --- |
| 1B | y w ey-Flip/+; act>y^+^>Gal4 UAS-GFP/+; UAS-dicer2/+ | 25°C |
|  | y w ey-Flip/+; act>y^+^>Gal4 UAS-GFP/+; UAS-dicer2/UAS-sdRNAi | 25°C |
| 1C | y w ey-Flip/+; act>y^+^>Gal4 UAS-GFP/+; UAS-dicer2/+ | 25°C |
|  | y w ey-Flip/+; act>y^+^>Gal4 UAS-GFP/+; UAS-dicer2/UAS-ykiRNAi | 25°C |
|  | y w ey-Flip/+; act>y^+^>Gal4 UAS-GFP/+; UAS-dicer2/UAS-sdRNAi | 25°C |
| 2A | pnr-Gal4 UAS-GFP/UAS-GFP | 25°C |
| 2B | y w UAS-dicer2 / +; pnr-Gal4/UAS-ykiRNAi | 25°C |
| 2C | y w ey-Flip/+; act>y^+^>Gal4 UAS-GFP/dpp-lacz; UAS-dicer2/+ | 25°C |
|  | y w ey-Flip/+; act>y^+^>Gal4 UAS-GFP/tsh-lacz; UAS-dicer2/+ | 25°C |
|  | y w ey-Flip/+; act>y^+^>Gal4 UAS-GFP/+; UAS-dicer2/+ | 25°C |
| 2D | y w ey-Flip/+; act>y^+^>Gal4 UAS-GFP/dpp-lacz; UAS-dicer2/UAS-sdRNAi | 25°C |
|  | y w ey-Flip/+; act>y^+^>Gal4 UAS-GFP/tsh-lacz; UAS-dicer2/UAS-sdRNAi | 25°C |
|  | y w ey-Flip/+; act>y^+^>Gal4 UAS-GFP/+; UAS-dicer2/UAS-sdRNAi | 25°C |
| 3A | y w ey-Flip/+; act>y^+^>Gal4 UAS-GFP/+; UAS-dicer2/+ | 25°C |
|  | y w ey-Flip/+; act>y^+^>Gal4 UAS-GFP/+; UAS-dicer2/UAS-sdRNAi | 25°C |
| 3B | w; Canton-S | 25°C |
|  | Diap1-2-lacZ | 25°C |
| 3C | y w ey-Flip/+; act>y^+^>Gal4 UAS-GFP/tub-Gal80^ts^; UAS-dicer2/UAS-sdRNAi | 18 → 29°C |
| 3D | y w ey-Flip/+; act>y^+^>Gal4 UAS-GFP/tub-Gal80^ts^; UAS-dicer2/UAS-sdRNAi | 29 → 18°C |
| 4A | y w ey-Flip/+; act>y^+^>Gal4 UAS-GFP/+; UAS-dicer2/+ | 25°C |
| 4B | y w ey-Flip/+; act>y^+^>Gal4 UAS-GFP/ UAS-hthRNAi; UAS-dicer2/+ | 25°C |
| 4C | y w ey-Flip act>CD2>Gal4/+; UAS-HthGFP/+ | 25°C |
|  | y w ey-Flip act>CD2>Gal4/+; UAS-HthGFP/UAS-sdRNAi | 25°C |
|  | y w ey-Flip act>CD2>Gal4/+; UAS-HthGFP/UAS-ykiRNAi | 25°C |
| 4D | y w ey-Flip/+; act>y^+^>Gal4 UAS-GFP/+; UAS-Yki/+ | 25°C |
|  | y w ey-Flip/+; act>y^+^>Gal4 UAS-GFP/+; UAS-dicer2/UAS-sdRNAi | 25°C |
| 5A | y w ey-Flip/+; act>y^+^>Gal4 UAS-GFP/+; UAS-dicer2/+ | 25°C |
|  | y w ey-Flip/+; act>y^+^>Gal4 UAS-GFP/+; UAS-Yki/+ | 25°C |
| 5B | y w ey-Flip/+; act>y^+^>Gal4 UAS-GFP/+; UAS-dicer2/UAS-sdRNAi UAS-Yki | 25°C |
|  | y w ey-Flip act>CD2>Gal4/+; UAS-Yki/UAS-sdRNAi | 25°C |
| 5C | y w ey-Flip act>CD2>Gal4/UAS-dicer2; UAS-Yki/UAS-hthRNAi | 25°C |
| 6A | y w ey-Flip/+; act>y^+^>Gal4 UAS-GFP/+; UAS-Hpo/+ | 18°C |
|  | y w ey-Flip/+; act>y^+^>Gal4 UAS-GFP/UAS-Wts | 25°C |
|  | y w ey-Flip/+; act>y^+^>Gal4 UAS-GFP/+; UAS-Myr-mats/+ | 25°C |
| 6B | ex^G1^ ft^GTV^ / ex^G1^ ft^fd^ | 25°C |
|  | y w ey-Flip/+; act>y^+^>Gal4 UAS-GFP/UAS-wtsRNAi ; UAS-dicer2/+ | 25°C |
| 6D | y w ey-Flip/+; act>y^+^>Gal4 UAS-GFP/UAS-wtsRNAi; UAS-dicer2/UAS-ykiRNAi | 25°C |
| S1B | y w ey-Flip/+; act>y^+^>Gal4 UAS-GFP/+; UAS-dicer2/+ | 25°C |
| S2A | y w ey-Flip/+; tub-gal80 FRT40A/FRT40A; act>y^+^>Gal4 UAS-GFP/UAS-ykiRNAi | 25°C |
| S2B | w; Canton-S | 25°C |
|  | y w ey-Flip act>CD2>Gal4/+; UAS-Yki/+ | 25°C |
|  | y w ey-Flip act>CD2>Gal4/+; UAS-Yki/UAS-hthRNAi | 25°C |
| S3A | y w ey-Flip/+; act>y^+^>Gal4 UAS-GFP/+; UAS-dicer2/UAS-ykiRNAi (N) | 25°C |
|  | y w ey-Flip/+; act>y^+^>Gal4 UAS-GFP/ UAS-ykiRNAi (C); UAS-dicer2/+ | 25°C |
| S3B | y w ey-Flip/+; act>y^+^>Gal4 UAS-GFP/ UAS-sdRNAi (N); UAS-dicer2/+ | 25°C |
|  | y w ey-Flip/+; act>y^+^>Gal4 UAS-GFP/+; UAS-dicer2/UAS-sdRNAi (N+C) | 25°C |
| S3C | y w ey-Flip/+; act>y^+^>Gal4 UAS-GFP/+; UAS-dicer2/UAS-hthRNAi (HMS01112) | 25°C |
| S3D | y w UAS-dicer2 / +; pannier-Gal4/UAS-ykiRNAi |  |
| S4 | y w ey-Flip/+; tub-gal80 FRT40A/FRT40A; act>y^+^>Gal4 UAS-GFP/+ | 25°C |
|  | y w ey-Flip/+; tub-gal80 FRT40A/FRT40A; act>y^+^>Gal4 UAS-GFP/UAS-sdRNAi | 25°C |
|  | y w ey-Flip/+; tub-gal80 FRT40A/FRT40A; act>y^+^>Gal4 UAS-GFP/UAS-ykiRNAi | 25°C |
| S5 | y w ey-Flip/+; act>y^+^>Gal4 UAS-GFP/+; UAS-dicer2/+ | 25°C |
|  | y w ey-Flip/+; act>y^+^>Gal4 UAS-GFP/+; UAS-dicer2/UAS-sdRNAi | 25°C |
| S6A | y w ey-Flip/+; act>y^+^>Gal4 UAS-GFP/tub-Gal80^ts^; UAS-dicer2/UAS-sdRNAi | 18 → 29°C |
| S7 | y w ey-Flip act>CD2>Gal4/+; UAS-yki/UAS-hthRNAi | 25°C |
| S8A | y w ey-Flip/+; act>y^+^>Gal4 UAS-GFP/+; UAS-dicer2/UAS-cycERNAi | 25°C |
|  | y w ey-Flip/+; act>y^+^>Gal4 UAS-GFP/+; UAS-dicer2/UAS-mycRNAi | 25°C |
|  | y w ey-Flip/+; act>y^+^>Gal4 UAS-GFP/+; UAS-dicer2/UAS-notchRNAi | 25°C |
| S8B | y w ey-Flip/+; act>CD2>Gal4/UAS-lacZ; UAS-ykiRNAi/+ | 25°C |
|  | y w ey-Flip/+; act> CD2>Gal4/UAS-P35; UAS-ykiRNAi/+ | 25°C |
| **not shown** | | |
|  | y w; ey-Gal4/+; UAS-dicer2/UAS-ykiRNAi | 25°C |
|  | y w; ey-Gal4/+; UAS-dicer2/UAS-sdRNAi | 25°C |
|  | y w ey-Flip/+; tub-gal80 FRT40A/FRT40A; act>y^+^>Gal4 UAS-GFP/UAS-Yki | 25°C |

(1) The RNAi lines TRiP# HMS00041 (yki-RNAi), TRiP# JF02514 (sd-RNAi), VDRC# 12763 (hth-RNAi) are shown in all panels except in Fig S3 (lines used as indicated in table).
