# Supplementary material for: Yki/YAP, Sd/TEAD and Hth/MEIS Control Tissue Specification in the Drosophila Eye Disc Epithelium
Source: PLoS One. 2011 Jul 19;6(7):e22278. doi: 10.1371/journal.pone.0022278 (PMC3139632; doi:10.1371/journal.pone.0022278)
Supplement: Table S2 — RNAi lines used in this work. (DOC) [file pone.0022278.s010.doc]

**Supplemental Table** S2: RNAi lines used in this work

| **Gene** | **RNAi ID # / Name** | **Target Region** | **Off- targets** | **References** |
| --- | --- | --- | --- | --- |
| ***sd*** | TRiP# JF02514 | aa 248-389 (Sd-RB) | NONE | 53,54 |
|  | Sd-RNAi^N^ | aa 36-169 (Sd-RB) | NONE | 13 |
|  | Sd-RNAi^C^ | aa 183-440 (Sd-RB) | NONE | 13 |
| ***yki*** | TRiP# HMS00041 | aa 48-55**^(1)^** | NONE | 53,54 |
|  | Yki-RNAi^N^ | aa 1-180**^(1)^** | NONE | 13 |
|  | Yki-RNAi^C^ | aa 285-418**^(1)^** | NONE | 13 |
| ***hth*** | VDRC# 12763 | aa 100- 214 (Hth-RC) | CG31204^(2)^ | 55 |
|  | TRiP# HMS01112 | aa 190-197 (Hth-RC) | NONE | 53,54 |
| ***wts*** | VDRC# 106174 | aa 654-780 (wts-PA) | CG17579^(2)^ | 37 |

(1) as per Yki aa sequence reported in Huang et al., 2005 [11].

(2) CG31204 encodes a protein of unknown function. CG17579 encodes Scabrous (a factor required for eye neurogenesis in the L3 disc, but with no known role in eye specification). Both RNAi lines have been previously validated for down-regulation of Hth and Wts, respectively, as per references provided.
