# Supplementary material for: Yki/YAP, Sd/TEAD and Hth/MEIS Control Tissue Specification in the Drosophila Eye Disc Epithelium
Source: PLoS One. 2011 Jul 19;6(7):e22278. doi: 10.1371/journal.pone.0022278 (PMC3139632; doi:10.1371/journal.pone.0022278)
Supplement: Table S3 — Relative strength of gene silencing. (DOC) [file pone.0022278.s011.doc]

| **Genotype:**  **eyflip; act>>Gal4 +** | **RNAi line** | **partial**  **transformation ^(1)^** | **strong**  **transformation ^(2)^** |
| --- | --- | --- | --- |
| 1X UAS-sdRNAi | TRiP# JF02514 | 48 (96%) | 2 (4%) |
| 2X UAS-sdRNAi | TRiP# JF02514 | 0 (0%) | 50 (100%) |
| 1 X UAS-ykiRNAi | TRiP# HMS00041 | 0 (0%) | 50 (100%) |
| 2 X UAS-ykiRNAi | TRiP# HMS00041 | 0 (0%) | 50 (100%) |
| 1 X UAS-hthRNAi | VDRC# 12763 | 38 (76%) | 12 (24%) |
| 2 X UAS-hthRNAi | VDRC# 12763 | 29 (58%) | 21 (42%) |

**Supplemental Table S3: relative strength of gene silencing**

(1) All flies displayed reduced heads with protruding eyes as in Fig. S6 phenotypes III & IV.

(2) All flies lacked heads, and residual eyes were either found in the thorax and or protruded from the anterior if the trunk region similar to Fig. S6 phenotype V.
